# Biomarkers and Detection Platforms for Human Health and Performance Monitoring: A Review

**DOI:** 10.1002/advs.202104426

**Published:** 2022-01-12

**Authors:** Daniel Sim, Michael C. Brothers, Joseph M. Slocik, Ahmad E. Islam, Benji Maruyama, Claude C. Grigsby, Rajesh R. Naik, Steve S. Kim

**Affiliations:** ^1^ Air Force Research Laboratory 711th Human Performance Wing Wright‐Patterson Air Force Base OH 45433 USA; ^2^ Research Associateship Program (RAP) the National Academies of Sciences, Engineering and Medicine Washington DC 20001 USA; ^3^ Integrative Health & Performance Sciences Division UES Inc. Dayton OH 45432 USA; ^4^ Air Force Research Laboratory Materials and Manufacturing Directorate Wright‐Patterson Air Force Base OH 45433 USA; ^5^ Air Force Research Laboratory Sensors Directorate Wright‐Patterson Air Force Base OH 45433 USA

**Keywords:** biosensor technologies, human health and performance biomarkers, human health and performance monitoring

## Abstract

Human health and performance monitoring (HHPM) is imperative to provide information necessary for protecting, sustaining, evaluating, and improving personnel in various occupational sectors, such as industry, academy, sports, recreation, and military. While various commercially wearable sensors are on the market with their capability of “quantitative assessments” on human health, physical, and psychological states, their sensing is mostly based on physical traits, and thus lacks precision in HHPM. Minimally or noninvasive biomarkers detectable from the human body, such as body fluid (e.g., sweat, tear, urine, and interstitial fluid), exhaled breath, and skin surface, can provide abundant additional information to the HHPM. Detecting these biomarkers with novel or existing sensor technologies is emerging as critical human monitoring research. This review provides a broad perspective on the state of the art biosensor technologies for HHPM, including the list of biomarkers and their physiochemical/physical characteristics, fundamental sensing principles, and high‐performance sensing transducers. Further, this paper expands to the additional scope on the key technical challenges in applying the current HHPM system to the real field.

## Graphical abstract

Detecting biomarkers with novel or existing sensor technologies is emerging as critical human monitoring research. This review provides a broad perspective on the state‐of‐art biosensor technologies for human health and performance monitoring, including the list of biomarkers and their physiochemical/physical characteristics, fundamental sensing principles, high‐performance transducers, and the key technical challenges in applying the current system to the real field.

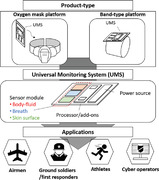

## 1 Introduction

Minimally invasive and noninvasive biomarker sensors have shown tremendous potential for human health and performance monitoring (HHPM).^[^ ^1^, ^2^, ^3^ ^]^ HHPM tools that report individuals' physical, psychological, and physiological readiness and ability to carry out assigned tasks successfully can play a significant role in many employment sectors including the military,^[^ ^4^ ^]^ first responders,^[^ ^5^ ^]^ and aerospace.^[^ ^6^, ^7^ ^]^ Conventional methods, such as medical examination, questionnaire‐based self‐reports, and cognitive tests, have been widely used to evaluate human statuses (i.e., physical fatigue, mental stress, and cognitive ability). However, these assessment methods are impractical to use in real‐time during operational situations and typically require highly trained personnel to evaluate. It is theorized that the most effective way to objectively assess human health and performance (HHP) in near‐real‐time is to observe biomarkers that are direct responses originating from the physiologic changes of an individual. For example, the steroid hormone cortisol is a well‐known temporal biomarker that is also produced as a response to psychological stressors. Therefore, when a person's bodily cortisol level increases, one can deduce that the subject is experiencing psychological stress. However, confounding this, is that cortisol also increases after meals, thus highlighting the need for integrated sensor networks.

**Figure 1:**
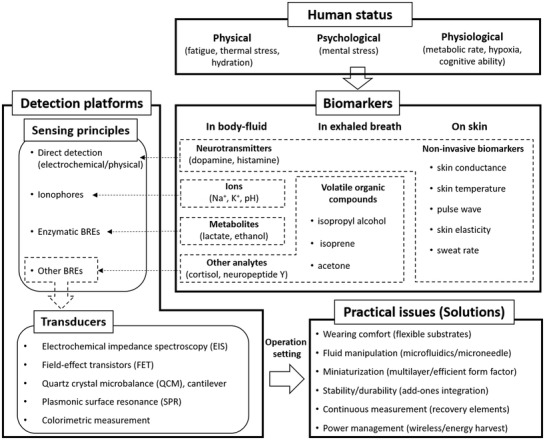
Human status monitoring through the biomarker detection strategies including biomarkers, sensing principles, transducers, and practical issues.

Monitoring a panel of biomarkers utilizing a minimally‐ or noninvasive biosensor would make it possible to assess these biomarker levels on‐site, both quantitatively and objectively. The discovery of biomarkers indicative of HHP is still in the research phase and limits the number and scope of targets that biosensor technologies can currently address. In practical applications, many issues associated with operational circumstances (i.e., mission/work task environments) remain to be addressed and represent obstacles for achieving accurate biosensor measurements. While several well‐organized review papers have addressed health‐relevant biomarker sensors mostly characterized in laboratory settings,^[^ ^8^, ^9^, ^10^, ^11^, ^12^, ^13^, ^14^, ^15^ ^]^ the novelty of this review is to outline sensor technologies specifically for HHPM with a focus on psychophysiological biomarkers and to address practical issues in the actual fields by discussing how to detect such biomarkers under various environments. Although it is not possible to include all recent publications, this review covers widely used and common technologies related to biomarker detection and sensing platforms (**Figure** **1**). First, the review introduces “human health and performance biomarkers (HHPBs)” found in several biological media detectable with a minimally invasive or noninvasive sensing platform. The review then outlines sensing principles and transducers to highlight how the sensing principles provide measurable signals over biomarker detections. Lastly, we address practical aspects of sensors that critically affect commercialization associated with HHPM and provide recent research progress to resolve such challenges.

## 2 Human Health and Performance Biomarkers

Biomarkers widely range in their appearance, secretion location, molecular weights, and physicochemical properties. We chose human body fluids, exhaled breath samples, and physical signatures as the representative biomarker types, and show a comprehensive list of these in **Table** **1**. This table provides an outlook of the range of biomolecules/analyte concentrations and measuments, aiming to help the readers glance into the scientific requirements and technical criteria for the corresponding biomarker sensor and monitor development. The four biomarker types: 1) biomarkers in body fluid (noninvasive), 2) biomarkers in body fluid (minimally invasive), 3) vapor‐phase biomarkers, and 4) noninvasive physical biomarkers on the skin, are described further to provide an insight into the recent research progress.

**Table 1:** Representative biomarkers reflecting the human status (* bpm: beat per minute, ** bpm: breaths per minute)

Biomarker type	Biomarkers	Reflecting human status (Note)	Measurand/concentration range (Note)
Biomarkers in body fluid	Na^+^	Hydration state^[^ ^244^ ^]^	>291 mmol h^−1^ (in sweat)^[^ ^244^ ^]^
	K^+^	Hydration state^[^ ^245^ ^]^	>200 mg h^−1^ (in sweat)^[^ ^245^ ^]^
	Lactate	Fatigue level^[^ ^246^ ^]^	<25 × 10^−3^ m (in sweat)^[^ ^195^ ^]^ <17.3 × 10^−3^ m (in blood), < 1.6 × 10^−3^ m (in saliva)^[^ ^247^ ^]^
	Cortisol	Psychological stress^[^ ^248^ ^]^	8.16–141.7 ng mL^−1^ (in sweat)^[^ ^249^ ^]^
	Dopamine	Psychological stress^[^ ^250^ ^]^	0.01 × 10^−6^ –1 × 10^−6^ m (in extracellular fluid)^[^ ^251^ ^]^
	Urea	Metabolism status^[^ ^252^ ^]^	100–250 mmol L^−1^ (in sweat)^[^ ^253^ ^]^
	Histamine	Acute stress^[^ ^254^ ^]^	>65 ng mL^−1^ (in blood)^[^ ^255^ ^]^
	Pyrrole	Psychological stress^[^ ^255^, ^256^ ^]^	>20 µg dL^−1^ (in urine)^[^ ^255^ ^]^
	Neuropeptide Y	Psychological stress^[^ ^257^ ^]^ Thermal stress^[^ ^258^ ^]^	>129 pm (in cerebral plasma)^[^ ^259^ ^]^
Vapor‐phase biomarkers	Acetone	Metabolic rate^[^ ^260^ ^]^	10–76 ppm (in exhaled breath)^[^ ^261^ ^]^
	Isoprene	Hypoxia/respiratory status^[^ ^262^ ^]^	274.9–474 ppb (in exhaled breath)^[^ ^263^, ^264^ ^]^
	Ethane	Psychological stress^[^ ^265^ ^]^	5–9 ppb (in exhaled breath)^[^ ^266^ ^]^
	Malondialdehyde	Cellular oxidation status^[^ ^267^ ^]^	30–130 nmol L^−1^ (in exhaled breath)^[^ ^267^ ^]^ 78.2–107.7 nmol L^−1^ (in exhaled breath)^[^ ^268^ ^]^
	Isopropyl alcohol	Toxic health (aerotoxic syndrome)^[^ ^32^ ^]^	50–250 ppb^[^ ^269^ ^]^
Noninvasive physical biomarkers on skin	Skin temperature	Thermal stress (by mean temperatures)^[^ ^37^ ^]^ Psychological stress (peripheral temperature)^[^ ^270^ ^]^	25–36 °C (on peripheral spot on skin)^[^ ^100^ ^]^
	Skin impedance/conductance	Psychological stress (on palm and volar wrist)^[^ ^41^ ^]^ Hydration state^[^ ^174^ ^]^	2–20 µS^[^ ^271^ ^]^
	Skin hardness	Thermal stress^[^ ^47^ ^]^	20–30 duro (shore 00)^[^ ^47^ ^]^
	Pulse wave (heart rate)	Physical and psychological stress^[^ ^41^, ^272^ ^]^	50–200 bpm* (0.83–3.66 Hz)^[^ ^108^ ^]^
	Sweat rate	Thermal stress^[^ ^55^ ^]^	0–110 g m^−2^ h (from no stress to strong heat stress)^[^ ^273^ ^]^
	Breathing rate	Psychological state^[^ ^274^ ^]^	4–65 bpm**^[^ ^275^ ^]^
	Blood pressure	Physical and psychological stress^[^ ^276^ ^]^	$\frac{>120\;\text{mm}\;\text{Hg}\;(\mathrm{systolic})}{>80\;\text{mm}\;\text{Hg}\;(\mathrm{diastolic})}$ ^[^ ^50^ ^]^

### 2.1 Biomarkers in Body Fluid (Noninvasive)

The gold standard for health‐based diagnostics is blood‐based, filtration‐free monitoring of biomarkers that regulate or are regulated by psychophysiological changes. However, direct insertion of sensors or devices into blood vessels is not practical for many reasons. Therefore, noninvasive biofluids (e.g., sweat, saliva, urine, and tears) are preferred pathways to sample biofluids. Many of these biomarkers either are signaling molecules such as hormones and cytokines, or are part of signaling pathways such as blood clotting markers. These biomarkers can range dramatically in terms of their phase/partitioning, size, charge density, and volatility. Aqueous‐phase analytes of interest vary from monoatomic ions, small molecules (e.g., steroid hormones and metabolites), and proteins (e.g., cytokines and chemokines). Neurotransmitters and hormones (both steroid and protein‐based) have been commonly looked at due to their ability to control and modulate physiological functions. Additionally, metabolites report on physiological activity and can be informative of biological processes. Urine detection is typically only used for point‐of‐care diagnostics for small molecules due to its variable dilution, low protein content, and periodic production. Therefore, for real‐time diagnostics, saliva, sweat, and tears are preferred biofluids for wearable sensing platforms. One of the existing challenges is that sweat, saliva, and urine have substantially decreased sample concentrations of typical protein biomarkers (<0.01 × compared to blood) due to filtration effects, especially for endogenous markers with HHP. Other biomarkers have unknown correlations; for example, it has been demonstrated that hormones found in saliva (cortisol, aldosterone, and testosterone) are currently unable to be correlated to concentrations in blood and serum.^[^ ^16^ ^]^ It has been hypothesized that sweat has levels of these hormones similar to concentrations of free hormones observed in the blood. However, many of the hormones of interest such as testosterone, have not yet been detected in eccrine sweat, which is more readily sampled and does not require collection on follicle‐ridden regions (i.e., armpits).

### 2.2 Biomarkers in Body Fluid (Minimally Invasive)

Sample variability due to the dilution of analyte levels from the filtration effect has led researchers to explore interstitial fluid (ISF), which is more invasive than monitoring excretory fluids. ISF has biomarker concentrations within an order of magnitude similar to that of blood/serum samples, making it a prime candidate for wearable biofluid sensors.

ISF exists primarily in the dermis, ≈1–4 mm deep from the skin surface,^[^ ^19^ ^]^ thus the most difficult to extract than other body fluids. ISF flows around skin physiological elements such as sweat glands and capillary vessels,^[^ ^17^ ^]^ considered the most biologically rich. Mass spectral data have suggested that at least 80% of proteins found in serum are also found in ISF.^[^ ^18^, ^19^ ^]^ Other studies performed on rats have suggested that the protein expression similarities may be more significant than 95%, with additional similarities in the transcriptome (>75%); the transcriptome is even more similar if we assume that ISF is a combination of biomarkers from plasma and blood (>90%). More importantly, the concentration of these proteins is similar to blood (substantially higher than in noninvasive body fluids), with a comparison ratio ranging between 1:2 and 2:1 (ISF:Blood) protein concentrations. ISF in some cases has been demonstrated to be enriched in biomarkers in some mammals. For example, in rats, while the proteome itself was highly similar between blood and ISF samples, exosome proteins are found in an order of magnitude higher concentrations in ISF.^[^ ^20^ ^]^ Identification of biomarkers in ISF and the development of additional sensors for ISF both suffer from an inability to reproducibly extract ISF and deliver sufficient volumes (>10 s of μLs) to collectors and devices piercing capillaries and thus extracting blood. To solve these challenges, many types of research have explored the use of fluid manipulation techniques (e.g., microneedles and microfluidics) that can increase the sample's extraction reliability and integrity.^[^ ^21^, ^22^, ^23^ ^]^

### 2.3 Vapor‐Phase Biomarkers

Breath analysis biomarkers include common gases such as O_2_, CO_2_ (hyperoxia/hypoxia), CO (inflammation),^[^ ^24^ ^]^ and NO (inflammation),^[^ ^25^ ^]^ as well as volatile and semivolatile organic compounds (VOCs and SVOCs). VOCs are defined as molecules with boiling points less than 250 °C, whereas SVOCs have boiling points between 250 and 400 °C. There are more than 1000 VOCs potentially offering abundant information about human status in exhaled breath. For breath VOCs and SVOCs, we observe hydrocarbons indicative of metabolic byproducts (i.e., acetone),^[^ ^26^ ^]^ isoprene,^[^ ^27^ ^]^ isopropyl alcohol (IPA),^[^ ^28^ ^]^ or fatty acid derivatives (i.e., pentane)^[^ ^29^ ^]^ that in some cases are indicative of inflammation (i.e., isoprostane).^[^ ^30^ ^]^ Substantial work is needed to correlate these biomarkers to the physiological state, but they remain promising.

Some more recent studies have begun to demonstrate that VOCs serve as promising biomarkers to assess HHP. For example, changes in breath isoprene levels can indicate hypoxia, as it has been found to have a close correlation to acute hypoxic stress.^[^ ^31^ ^]^ IPA levels affect cognitive ability of the individual^[^ ^32^ ^]^ as IPA exposure contaminates breathing air and acts as an anesthetic to the central nervous system. Acetone is related to metabolic rate;^[^ ^33^ ^]^ The profile of acetone exhalation aligned well with the results from clinical studies of serum glucose regulation (metabolic activity). Toluene is related to chronic systemic intoxication;^[^ ^34^ ^]^ It causes mucous membrane irritation, decrements in central nervous system function and endocrine disruption. The current challenge for VOC detection is the ultralow concentration of biomakers in the exhaled breath. Due to the rapid movement of inhalation and exhalation (≈6 L min^−1^), the concentrations of breath analytes range within the ppt to ppb range requiring highly sensitive sensor modalities.

### 2.4 Noninvasive Physical Biomarkers on Skin

Noninvasive physiological measurements on human skin surfaces have been utilized mainly in clinical sectors due to their simplicity in measurement and ease of access. The skin physiological data have provided preliminary health information to the clinicians within a short period of time. For example, clinical sectors have utilized these physiological signals to monitor patient's vital signs in real‐time during surgery. A number of skin physiological readouts, such as temperature, conductance, elasticity, cardiac pulse wave, etc., can serve as biomarkers,^[^ ^35^, ^36^ ^]^ reflecting thermal/psychological stress levels. This section presents a few examples of physiological signals serving as biomarkers that can be noninvasively and physically measured on a skin surface.

#### 2.4.1 Mean Skin Temperature and Skin Conductance

Mean skin temperature (MST) has been used as an indicative parameter for thermal comfort and stress levels^[^ ^37^, ^38^ ^]^ due to its close correlation to core body temperature. MST is calculated from 3 to 21 skin sites depending on the MST models. While the requirement of multiple temperature sensors is rather cumbersome for real‐time human status measurement, the MST is one of the most accessible and cost‐effective biomarkers to detect. The skin conductance is an excellent alternative to MST, reflecting thermal comfort levels and psychological stress levels depending on the skin region where the electrical probe is in contact. Skin conductance can be correlated to several physiological changes, such as sweat secretion.^[^ ^39^ ^]^ The electrical activity of palmar and plantar skin has shown to be strongly related to psychological stress.^[^ ^40^ ^]^ For example, skin conductance on the palm and volar wrist was positively related to chronic or acute stress levels.^[^ ^41^ ^]^ In the psychophysiology study, a variation in skin conductance change was shown to be an essential indicator of a sudden emotional change.^[^ ^42^, ^43^ ^]^ While skin conductance is easy to measure (similar to skin temperature), one is required to select the dermal area carefully and account for any motion artifacts due to the inherent nature of electrical measurement vulnerable to external noise sources (e.g., body movement and muscle activity).^[^ ^44^ ^]^

#### 2.4.2 Mechanical Properties of Skin

Mechanical properties of skin (e.g., skin elasticity and skin hardness) are widely used in dermatology and cosmetology to assess skin age or skin diseases.^[^ ^45^, ^46^ ^]^ A recent paper reported that skin hardness could serve as a physiological biomarker indicating human thermal status.^[^ ^47^ ^]^ The study focuses on hair follicles‐connected arrector pili muscles^[^ ^48^ ^]^ that contract and expand depending on human thermal status, providing measurable changes in skin hardness. Another paper demonstrated that morphological changes in arrector pili muscles known as “goose bump” could serve as a biomarker indicating sudden emotional changes.^[^ ^43^, ^49^ ^]^ Although such mechanical biomarkers are detectable, a sensitive detection scheme is required due to the narrow and low range of measurand.

#### 2.4.3 Arterial Pulse Wave

The arterial pulse wave is a physiological phenomenon of the arterial system during blood circulation, including systolic and diastolic blood‐flowing activities.^[^ ^50^, ^51^, ^52^ ^]^ Arterial pulse wave has been one of the most representative biomarkers for estimating human physical and psychological states. The arterial pulse wave can indicate heart rate, which is essential information for human physical activity. The arterial pulse wave data is transformed to heart rate variability (HRV), defined as variations in time intervals between peaks of arterial pulsation. HRV is controlled by the autonomic nervous system, representing psychological stress changes and stress vulnerability.^[^ ^53^ ^]^ Cutaneous blood flow relates to the amount of blood, whereas arterial pulse wave is related more closely to a frequency domain. As the thermoregulation process in the human body controls blood flow, cutaneous blood flow in peripheral body regions such as fingertips and toes is closely related to the human thermal status. When a person feels hot or cold, peripheral blood flow increases or decreases to control core body temperature. While such noninvasive skin biomarkers can serve as a relatively straightforward tool to withdraw human status levels, they are susceptible to motion artifacts.

#### 2.4.4 Sweat Rate

Sweat rate, the amount of water mass generated from the skin within a specific time and area,^[^ ^54^ ^]^ can serve as a thermal status‐related biomarker. The sweat rate has shown a linear relationship with core body temperature,^[^ ^55^ ^]^ implying its close relationship to human thermal comfort status. The sweat rate has the advantage of being measured on a small part of the skin and being insensitive to the motion artifact. However, sweat rate can only be applicable for the situations when people feel warm or hot. The reduction of sweat secretion in cold or cool weather inhibits the precise sweat rate measurement.

The noninvasive skin physical biomarkers enable a straightforward measure of human status in real‐time. Thus, it is crucial to establish in‐vivo measuring schemes to capture such biomarkers appropriately. Skin sensor research has employed various sensing principles suitable for wearables to detect these physical biomarkers from the skin surface efficiently.

## 3 Biomarker Sensing Principles

**Figure 2:**
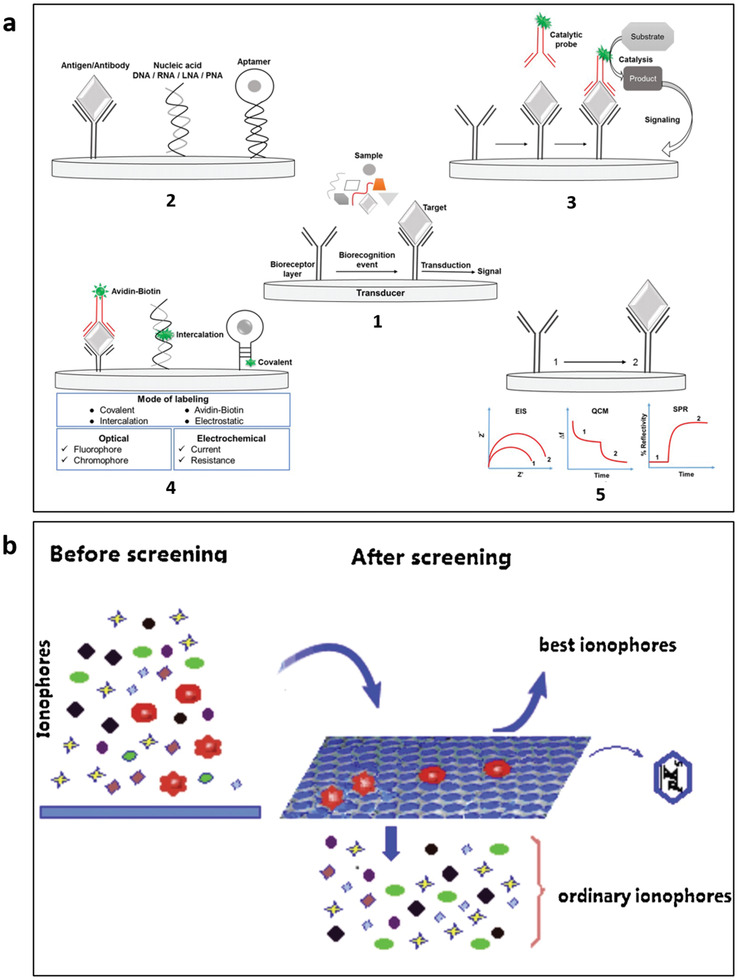
Biochemical sensing principles for detecting biomarker in body fluid. a) (1–5) Biorecognition elements. Reproduced with permission.^[^ ^56^ ^]^ Copyright 2017, IOP Publishing. b) Ionophores. Reproduced with permission.^[^ ^59^ ^]^ Copyright 2016, Elsevier.

Detection of biomarkers relies on determining changes in optical, mechanical, electrical, or electrochemical properties. Detection of analytes in biofluids is typically based on immunoprecipitation. Biorecognition elements (BREs) (**Figure** **2**a.1‐2)^[^ ^56^ ^]^ are used to recognize the analytes. When enzymes are used, a biochemical reaction can generate a byproduct proportional to the analyte, providing another pathway toward detection (Figure 2a.3). Of the sensing modalities available, electrical or electrochemical interrogation is often demonstrated due to the availability of mature technology for both generation and detection of electrical pulses, where sensor outputs correspond to analyte concentration including current, resistance, or potential changes (Figure 2a.4). Fluorophore‐ and chromophore‐labeled detections are also available for optical measurement. In some cases, a reaction can then be leveraged to generate or amplify a signal using transducers (Figure 2a.5). Several physical sensing principles based on optical, electrical, or mechanical properties detect the physical biomarkers. The following sections discuss the interplay between specific classes of biomarkers and sensing mechanisms for selective detection (**Table** **2**).

**Table 2:** Biomarker sensing principles and their characteristics

Sensing principles	Detectable biomarkers	Characteristics/notes
Direct detection of redox active analytes	Redox‐active small molecule Neurotransmitters (e.g., serotonin, epinephrine, norepinephrine, serotonin, dopamine) Specific hormone (e.g., melatonin)	Detection using direct oxidation/reduction Detection without biorecognition element (BRE)
Potential‐based measurements	Ions (e.g., Na^+^, K^+^, Ca^2+^) pH	Selective detection using ionophores‐based ion‐selective electrodes
Current‐based measurement	Metabolites (e.g., lactate, glucose, ethanol)	Use of enzyme BREs to detect byproduct of catalytic conversion of biomarkers Need of compensations for the salinity, pH, or temperature for accurate measurement
Impedimetric measurements	Cortisol, Neuropeptide Y Volatile organic compounds (e.g., acetone, isopropyl alcohol, and isoprene)	Use of immunoprecipitation BREs (e.g., protein, antibody) Challenges with reliable biofunctionalization, thermal stability, and shelf‐life
Direct detection of noninvasive physical biomarkers	Skin temperature/conductance Mechanical properties of skin Blood circulation Sweat rate	Measured by several conventional electrical, thermal, mechanical, optical, or fluidic sensing principles

### 3.1 Direct Detection of Redox‐Active Analytes

A limited number of redox‐active biomarkers exist in the body and can be detected without the addition of a BRE. They, instead, are detected directly by direct oxidation/reduction. These are small molecules and include the neurotransmitters of serotonin, epinephrine, norepinephrine, serotonin, dopamine, and specific hormones such as melatonin. Typically, these molecules contain substituted conjugated double bond systems (e.g., catechol) that allow for more labile transfer of electrons at specific electrochemical potentials. The potential of the oxidation/reduction then informs on the species, which can be determined by either square wave voltammetry (SWV) or fast scan cyclic voltammetry. To increase local concentrations at the electrodes, the sensor can be coated in negatively charged matrices that absorb the positively charged neurotransmitters. In wearable form, detection of norepinephrine has been demonstrated using a smart‐phone based SWV system^[^ ^57^ ^]^ leveraging Bluetooth communication with the reduction in size of potentiostats in conjunction with the increase in computational power in smart phones.^[^ ^58^ ^]^

### 3.2 Potential‐Based Measurements for Detection of Ions Using Ionophores

Ions, by their nature, have a charge and thus can change the electrostatic environment upon binding to a BRE. Ionophores are BREs that recognize ions. Ionophores are primarily hydrophobic, allowing them to embed into hydrophobic membranes while retaining a polar binding pocket with a shape and charge density enabling selectivity to ions of interest (Figure 2b).^[^ ^59^ ^]^ Detectable ions include oxygen‐rich functional groups such as ketones, ethers for positively charged ions, amine groups for negatively charged ions, and other reactive functionalities.^[^ ^60^, ^61^ ^]^ Numerous ionophores have been identified for various ions of interest, each with excellent selectivity toward the ions they bind. Commercially available ionophores have generally been demonstrated to have a selectivity of 2–3 orders of magnitudes toward the ion of interest.^[^ ^62^ ^]^

Most papers in the literature demonstrating real‐time biochemical analysis have used these ionophores in potentiometric sensors. The binding of an ionic analyte to the ionophore creates a charge separation and a measurable potential. Ion‐selective electrodes (ISEs) have been used for measurement of sodium (Na^+^),^[^ ^12^, ^63^, ^64^, ^65^, ^66^ ^]^ potassium (K^+^),^[^ ^12^, ^65^, ^66^ ^]^ calcium (Ca^2+^),^[^ ^67^ ^]^ and pH.^[^ ^67^ ^]^ In an exemplary case, the feedback provided from these ISEs has been used to correct the output signal of enzymatic sensors.^[^ ^68^ ^]^ ISEs have been incorporated successfully into microfluidic channels or patches in multiple cases, enabling temporal resolution.^[^ ^65^, ^68^, ^69^ ^]^

### 3.3 Current‐Based Measurements for Detection of Metabolites Using Enzymatic BREs

Enzymes are among the most abundant BREs in nature for both multicellular and unicellular organisms. They can also be engineered for enhanced stability and/or turnover rate through genetic engineering.^[^ ^70^ ^]^ For biosensors, enzymes commonly show that the by‐product of catalytic conversion of the analyte is capable of being detected (Figure 2a.3). Enzymatic sensors can be commonly classified into three generations based on the mechanism of electron transfer, and thus the compounds detected.^[^ ^71^ ^]^ 1st generation sensors use oxygen as the electron acceptor from the enzymatic cofactor (most commonly NADH, FADH_2_), and thus directly monitor the formation of hydrogen peroxide. 2nd generation sensors leverage a redox mediator, most commonly Prussian Blue, as the electron acceptor from the enzymatic cofactor. 3rd generation sensors create a conductive matrix in close enough proximity to the cofactor to enable direct electron transfer and detection; in many cases, this requires deglycosylation of the enzyme.^[^ ^72^, ^73^ ^]^ In alternative enzymatic detection schemes, the byproduct of an enzymatic reaction can be directly detected (such as when thiols are produced) or can be detected after a second enzymatic reaction. The second approach has been done for measuring acetylcholine, where the molecule is first cleaved to form choline plus acetate and then further oxidized to betaine aldehyde producing a redox‐active hydrogen peroxide molecule.^[^ ^74^ ^]^

Currently, wearable enzymatic sensors have demonstrated detections of lactate using lactate oxidase (LOx), FADH_2_ cofactor,^[^ ^75^ ^]^ glucose using glucose oxidase (GOx), FADH_2_ cofactor,^[^ ^68^, ^75^ ^]^ and ethanol using alcohol oxidase, FADH_2_ cofactor.^[^ ^76^ ^]^ These sensing demonstrations have been made using sweat derived through physical exertion and iontophoretic stimulation of sweat using a biochemical agent. In a more recent study, pH, temperature, and humidity were compensated to sensing signals to enhance the accuracy of the sensor.^[^ ^68^ ^]^ In most second‐generation sensors, Prussian blue has served as the redox mediator.

While these initial proof of concept tests are promising, multiple problems have yet to be fully solved to confound sensor outputs for enzymatic measurements. For example, enzymes are known to have their activity impacted by salinity, pH, and temperature. For sampling interstitial fluid or blood, changes in salinity and pH are not observed due to the homeostasis of the fluid composition with respect to these parameters. However, for excretory fluids such as sweat and saliva, simultaneous measurement and compensation for the salinity, pH, and temperature is imperative to ensure accurate measurements. Additionally, enzymatic kinetics are highly flow‐rate dependent, especially for high‐turnover enzymes that locally deplete the target substrate. Sweat can vary with regards to flow rate by over two orders of magnitude. Therefore, monitoring of flow rate is imperative to proper enzymatic sensor function for sweat.^[^ ^77^, ^78^, ^79^ ^]^

Enzymes are prone to proteolysis and unfolding over time, especially upon losing their redox cofactors FADH_2_ and NADH. These cofactors have been shown to have low nm to high pm affinity for their substrates, with NADH cofactors bound more weakly than FADH_2_ cofactors. Sweat typically contains proteases that are important for the defensive characteristics of the skin barrier. Therefore, time compensation for loss of enzymatic activity must be accounted for or prevented through incorporating stabilization strategies on the immobilized enzyme onto a solid‐state matrix, such as a metal electrode.

### 3.4 Impedimetric Measurements Based on Immunoprecipitation BREs

Immunoprecipitation uses BRE to bind its analyte of interest (such as a protein or steroid hormone). The binding event triggers an electrochemical signal using redox‐active transducers (Figure 2a.5). In nature, antibodies and membrane‐associated receptor proteins can rapidly sense and respond to multiple targets, detect environmental stimuli, and operate efficiently in complex biofluids by using an assortment of highly specialized biomolecules with high binding affinities and precise biological structures. Similarly, BREs have been widely used in biosensors to mimic the binding activity and function of naturally occurring biomolecules.^[^ ^80^ ^]^ The BREs include 1) biomolecules derived from natural sources (i.e., antibodies, odorant‐binding peptides), 2) peptides and DNA/RNA aptamers identified from large combinatorial libraries (>10^9^ random sequences) including modified and nonnative nucleic acids, and 3) biomolecules designed in silico. Recently, in silico approaches were successfully used to increase the binding affinity of a troponin‐binding peptide (*K*_d_ = 0.23 × 10^−9^ m) to detect human cardiac troponin. The screening of combinatorial libraries has revealed new and unique target‐specific peptide sequences against viruses, bacteria, and HHPB.^[^ ^81^ ^]^ Consequently, for wearable sensors, the use of BREs is highly appealing to enhance and augment the overall performance and functionality of devices, achieve lower detection limits of targets, obtain greater target specificity and selectivity, and increase sensor response times, and eliminate fewer false positives. More specifically, fluorinated RNAs or hydrophobically modified DNAs^[^ ^82^, ^83^, ^84^, ^85^ ^]^ have provided higher selectivity and sensitivity, enabling lower limits of detection.

Many high‐affinity receptors are membrane proteins or membrane‐associated proteins, as they bind, creating signaling cascades critical to intercellular communications.^[^ ^86^ ^]^ Membrane proteins are inherently challenging to synthesize as they require screening for detergents to solubilize and stabilize the membrane protein to maintain its functionality. Even then, the stability of the membrane protein and sensitivity to external conditions present challenges.^[^ ^87^ ^]^ Therefore soluble proteins are preferred as protein receptors.

The traditional soluble protein BRE is the antibody, which is 150 kDa and is produced from recombining exon fragments to identify sequences that bind to the receptor of interest. However, size and stability issues have caused the exploration of smaller fragments. Nanobodies or single domain nanobodies are simply subfragments of antibodies from the variable chain region,^[^ ^88^ ^]^ retaining a high level of specificity to their substrate. Whilst numerous studies have used protein BREs in electrochemical sensors, very few have demonstrated them in a wearable platform. Most recently, cortisol was measured in graphene utilizing a cortisol antibody as a receptor for a competitive assay. An antibody was grafted onto the graphene surface, and both free and HRP modified cortisol competed for the antibody binding site using a “reagent‐added” scheme.^[^ ^89^ ^]^ This schematic used for offline analysis could still be deployed into a wearable device.

The integration and effectiveness of current BREs with wearable devices suffer from multiple challenges to date. These include achieving reliable and reproducible biofunctionalization of wearable device components and complementing material surface chemistries with BREs. Also, there is a need to increase the thermal stability and shelf‐life of BREs to extend the operation of wearable devices. To overcome biofunctionalization issues, BREs have been engineered for material binding by the addition of graphene‐ and gold‐binding peptides,^[^ ^90^, ^91^, ^92^, ^93^ ^]^ synthesis of thiolated aptamers, and cysteine modified peptides,^[^ ^94^, ^95^ ^]^ and by the introduction of ligands or cross‐linkable groups for immobilization to sensing material.^[^ ^96^, ^97^ ^]^ For example, carbon nanotubes were biofunctionalized with an odorant‐binding peptide from honeybees by creating a fusion peptide with a carbon nanotube binding peptide to detect trinitrotoluene.^[^ ^98^ ^]^ The use of transducers for converting binding events into measurable signals will be discussed in Section 4.

### 3.5 Direct Detection of Noninvasive Physical Biomarkers on Skin

**Figure 3:**
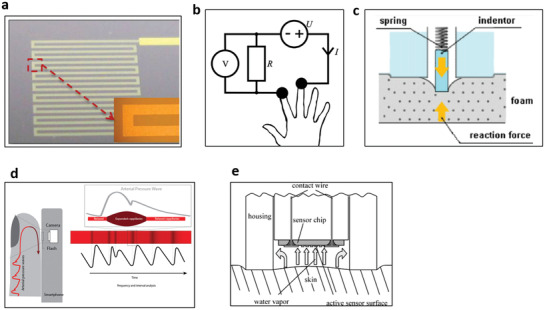
Physical sensing principles for measuring various biomarkers on skin. a) Resistance temperature detector (RTD) for skin temperature. Reproduced with permission.^[^ ^92^ ^]^ Copyright 2012, IOP Publishing. b) 2‐wire conductance measuring principle for skin conductance. Reproduced with permission.^[^ ^102^ ^]^ Copyright 2013, Elsevier. c) Indentation method for skin hardness. Reproduced with permission.^[^ ^105^ ^]^ Copyright 2006, IOP Publishing. d) Photoplethysmography (PPG) for pulse wave. Reproduced with permission.^[^ ^106^ ^]^ Copyright 2017, JMIR Publication. e) Closed chamber method for sweat rate. Reproduced with permission.^[^ ^110^ ^]^ Copyright 2008, Elsevier.

Noninvasive physical biomarkers on the skin can be measured by several conventional electrical, thermal, mechanical, optical, or fluidic sensing principles depending on the target biomarkers. The direct biomarker measurements on the skin need to comply with the product biocompatibility and safety standards and use only minimally chosen measurable skin parameters as sensing stimuli. This section briefly describes representative sensing principles for a few such physical biomarkers satisfying above requirements (**Figure** **3**).

Skin temperature measurements mainly utilize a thermocouple or a resistance temperature detector (RTD) due to their benefit of simplicity and manufacturability as a miniaturized platform. The thermocouple method^[^ ^99^ ^]^ consists of two different metal wires where one end is opened (physically disconnected) and the other end is electrically connected. The voltage difference between the metal wires occurs depending on the temperature at the connected end spot. The other open ends are used as electrodes to capture the voltage difference. An RTD utilizes a single metal material where the resistance of the metal is changing depending on the temperature (Figure 3a).^[^ ^100^, ^101^ ^]^ Commonly used metals are platinum and copper due to their high temperature coefficient of resistance.

Meanwhile, skin conductance measurements mainly utilize the 2‐wire resistance principle (Figure 3b),^[^ ^102^ ^]^ which is the simplest and easiest way to measure epidermal resistance by Ohm's law (resistance expressed by voltage divided by current). By applying a constant voltage to a specific skin region, the measured current can provide a resistance value indicating the skin conductance value. It is also possible to detect skin impedance by measuring AC profile to provide skin depth information. Skin conductance measurements have specific guidelines in terms of size and distance of electrodes as well as the skin interface method used to ensure reliability and accuracy.

Measurement of skin mechanical properties requires the careful design of the sensors since the skin is highly elastic and is not rigid. Thus, the skin sensor needs to record the skin modulus at much higher sensitivity than a typical solid mechanical characterization sensor. The most widely used principles to measure skin mechanical property are based on suction method^[^ ^103^ ^]^ and indentation method.^[^ ^104^ ^]^ The suction method applies negative pressure to a specific region of the skin surface to observe pressure‐induced skin deformation. Increased deformation indicates more elastic skin properties and vice‐versa. The indentation method (Figure 3c)^[^ ^105^ ^]^ uses sharp indenters to push the skin surface allowing for the detection of repulsive force from skin.^[^ ^104^ ^]^ This force is produced due to the response from indentation where the higher skin hardness provides the stronger the repulsive force.

For the indirect and noninvasive estimation of blood circulation conditions, arterial pulse waves can be measured using either optical principles using photoplethysmography (PPG) or mechanical principles using a piezoelectric membrane. PPG utilizes two components, a light source and a photodetector (Figure 3d).^[^ ^106^ ^]^ When the light source exposes lights to the skin, the reflected or transmitted light density changes depending on blood flow. The photodetector then captures the change in light density which directly reflects the arterial pulse wave. Piezoelectric membranes detect pressure differences in blood vessels between systolic and diastolic events where the pressure differences cause deflection of the membrane.^[^ ^107^ ^]^ Depending on the deflection, piezoelectric membranes generate voltages producing pulse wave signals. Since a normal blood pressure range sits between 40 and 120 mmHg at the frequency of 0.83–3.66 Hz,^[^ ^108^ ^]^ piezoelectric membranes should be sensitive with a fast response time for deflection.

Sweat rate measurement generally focuses on the amount of sweat evaporated from the skin surface.^[^ ^109^ ^]^ Sweat rate measurements use humidity collecting chambers and humidity sensors to measure the amount of evaporated sweat, water vapor. The sweat rate parameter is obtained by the change in humidity when the humidity chamber collects water evaporated from the skin. Dramatic changes in humidity mean an increased rate of sweat production (Figure 3e).^[^ ^110^ ^]^ There are a number of sweat rate measurement types, such as open‐chamber type and closed‐chamber type, where open‐chamber type is more straightforward but less stable, and close‐chamber is more stable but needs additional components to ventilate the chamber regularly.^[^ ^111^ ^]^ Recently, microfluidics‐based sweat rate measurements^[^ ^112^, ^113^ ^]^ have been developed. Capillary force results in sweat on the skin into a microchannel, and the total volume of sweat is calculated based on the channel length filled with water. Microfluidics‐based sweat rate measurements have become a promising platform due to their compatibility with biochemical sweat sensing.

## 4 Transducers

A transducer is a device that converts energy from one form to another. BRE‐based biomarker sensors (typically for label‐free BREs) require appropriate transducers to convert binding events between receptors and targets into measurable signals. As BRE research has emerged as a promising technology for selective sensing, the selection of transducer and its specific design dominantly determine the key features of the sensing platform as a wearable or portable monitoring system in terms of a type of signal output and size of the overall platforms. Therefore, understanding the principle and fundamental structure of transducers is important to design BRE‐based sensor platforms. Among several types of transducers, we choose the most broadly used transducers that include 1) electrochemical impedance spectroscopy/square wave voltammetry, 2) field‐effect transistors, 3) quartz crystal microbalances and cantilevers, 4) enhanced surface plasmon resonance, and 5) colorimetric principles. The following sections discuss working principles and recent advances of these transducers that have been utilized for human status monitoring applications (**Table** **3**).

**Table 3:** Transducers and their characteristics

Transducers	Principles	Characteristics (pros/cons)
Electrochemical impedance spectroscopy/square wave voltammetry	Use of AC waveform to interrogate the real and imaginary impedance	Sensitive to a variety of targets (e.g., proteins, small molecules, oligonucleotide) Need of improving miniaturization, drift, and fouling, drift, and fouling
Field‐effect transistors	Electrostatic modulation of charge carriers in the conduction channel (semiconductor or semimetal)	Low power consumption, miniaturization, continuous measurement Sensitivity variation under ions in an electrolyte
Quartz crystal microbalances/ cantilevers	The capture of mass variation in a specific area by detecting the changes in the frequency	Sensitive to target detection, continuous measurement Stability/durability/response time and reproducibility
Enhanced surface plasmon resonance	Use of plasmonically active metals responding to changes in the local surrounding environment from a binding event near the metal surface	Label‐free real‐time measurement, low cost, high sensitivity Miniaturization requirements of components (spectrometer, light source)
Colorimetric measurement	Use of colorimetric materials, including color responsive dyes and metal nanoparticles that change their colors in response to biomarkers	Straightforward and cost‐effective Image process required and loss of time resolution

### 4.1 Electrochemical Impedance Spectroscopy and Square Wave Voltammetry

**Figure 4:**
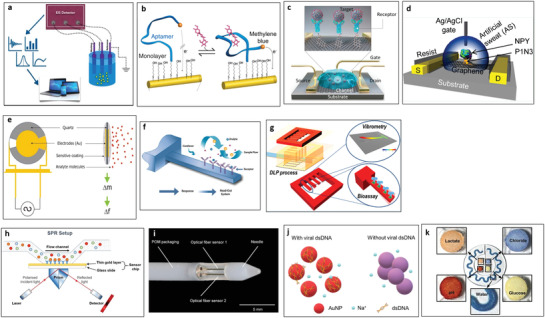
Transducers for biochemical sensing. a) Electrochemical impedance spectroscopy (EIS). Reproduced with permission.^[^ ^114^ ^]^ Copyright 2018, MDPI. b) Sensing efficacy improvement using antifouling self‐assembled monolayers for the real‐time biomarker monitoring in an ambulatory rat. Reproduced with permission.^[^ ^122^ ^]^ Copyright 2017, National Academy of Sciences. c) Field‐effect transistors (FETs). Reproduced with permission.^[^ ^127^ ^]^ Copyright 2020, American Chemical Society. d) Graphene‐based FETs characterized in physiologically relevant environments (an artificial sweat with various ionic concentrations of the electrolyte). Reproduced with permission.^[^ ^135^ ^]^ Copyright 2020, American Chemical Society. e) Quartz crystal microbalance (QCM). Reproduced with permission.^[^ ^141^ ^]^ Copyright 2004, CIGR Journal. f) Cantilever. Reproduced with permission.^[^ ^148^ ^]^ Copyright 2012, Elsevier. g) A 3D printed cantilever platform to overcome reproducibility and fabrication complexity issues. Reproduce with permission. Copyright 2017, American Chemical Society. h) Surface plasmon resonance (SPR). Reproduced with permission.^[^ ^149^ ^]^ Copyright 2002, Springer Nature. i) A plasmonic sensing probe with tilted fiber Bragg grating imprinted on optical silica fibers. Reproduced with permission.^[^ ^161^ ^]^ Copyright 2019, Elsevier. j) Colorimetric measurement. Reproduced with permission.^[^ ^167^ ^]^ Copyright 2020, Elsevier. k) Microfluidic colorimetric detection reservoirs for the determination of multiple biomarker concentrations. Reproduced with permission.^[^ ^164^ ^]^ Copyright 2016, AAAS.

Impedance‐based biosensors (**Figure** **4**a)^[^ ^114^ ^]^ rely on the use of AC waveforms to interrogate the real and imaginary impedance of a given chemical system.^[^ ^115^ ^]^ Impedance sensors have been deployed for a variety of targets (e.g., proteins,^[^ ^116^ ^]^ small molecules,^[^ ^117^, ^118^, ^119^ ^]^ and oligonucleotides)^[^ ^120^ ^]^ where both the redox reporter was in solution and directly attached to the BRE. For sensing applications, reagent‐less detection is preferred as it mitigates the requirement to add and partition a reagent, circumventing the need for complicating microfluidics. However, reagent‐less detection often requires selecting structure‐switching aptamers and re‐engineering aptamers.^[^ ^121^ ^]^

To date, little work has been done on in vivo/wearable sensors for large analytes. One of the most promising recent studies measured the aminoglycoside kanamycin in an ambulatory rat.^[^ ^122^ ^]^ This study developed multifrequency techniques to minimize the impact of drift, demonstrating antifouling techniques such as the filtering of proteins. Other promising work has demonstrated the efficacy of these sensors using anti‐fouling self‐assembled monolayers (Figure 4b),^[^ ^123^ ^]^ suggesting that this platform is translatable to a wearable device. Electrochemical impedance spectroscopy (EIS) and SWV have been demonstrated recently on flexible platforms using interdigitated electrodes and an integrated sensor with interdigitated wedges.

The development of BREs that are compatible with this technique is the primary challenge to implementing these sensors in sweat, saliva, and ISF. The concentration of analytes that are preferred to be detected using impedance‐based methodologies ranges drastically, from micromolar concentrations for select hormones to sub‐picomolar concentrations for most protein biomarkers. The development of high‐affinity aptamers with dissociation constants at time scales consistent with real‐time measurements will be critical for long‐term applicability and deployment. Additionally, pH variability needs to be addressed as pH^[^ ^124^, ^125^, ^126^ ^]^ levels impact the binding event and the most commonly used methylene blue reporter tag.

### 4.2 Field‐Effect Transistors

A field‐effect transistor (FET) (Figure 4c)^[^ ^127^, ^128^ ^]^ became popular in the 1980s^[^ ^129^ ^]^ as it could offer rapid electrical detection of charged molecules (targets) in a label‐free manner. FET‐based sensors promised low power consumption, miniaturization, portability, and the potential for on‐chip integration for sensing targets with a positive or negative charge. Although initial work on FETs started with conventional planar FETs,^[^ ^129^ ^]^ nonplanar geometries such as nanowires and nanotubes^[^ ^130^, ^131^ ^]^ have demonstrated better sensitivity and soon became more attractive for FET development. Generally, the sensitivity of FET depends on electrostatic modulation (using an electrolyte gate) of charged carriers in the conduction channel (which can be a semiconductor or semimetal) by the charges present in the target. Such modulation can be detected by flowing current through the conduction channel using source and drain electrodes. For a fixed number of charges in the target, an FET will show a higher sensitivity when there is an equivalent number of charged carriers in the channel. Therefore, target biomarkers can be sensed at a very low concentration (e.g., pm) using dimensionally constraint FET channels with large surface areas and shallow channel thickness as available in FET‐based sensors made with nanowire, nanotube, or monolayers of 2D materials like graphene and MoS_2_. Sensitivity in such a sensor can be increased by reducing the free carriers by introducing defects in the channel or forming a channel whose conduction is near the percolation threshold for conduction. In addition to sensitivity, the dimension of the channel also affects the response time measured in an FET. Generally, a low‐dimension channel requires a shorter response time due to the smaller diffusion time needed for targets to reach a smaller dimension channel surface.^[^ ^131^ ^]^ Measurement of electrical response for an FET is generally performed in DC mode by applying a DC voltage across two electrodes (source and drain) that connect the channel.^[^ ^129^, ^130^ ^]^ Alternately, a heterodyne AC signal and lock‐in amplifier can be used to measure the response.^[^ ^132^, ^133^ ^]^ Sensor response in DC mode is measured by monitoring either the voltage shift at a particular current level or the current change at a particular voltage. For voltage shift estimations, quantities like threshold voltage shift (for conventional FET) and Dirac voltage shift (for graphene FET) are often used. The magnitude of these voltage shifts depends on the electrostatic interactions between the target and charged carriers in the channel and are estimated using electrochemical analysis of the liquid‐solid interface present at the sensing surface of the FET. The Gouy–Chapman–Stern model^[^ ^134^, ^135^ ^]^ is advantageous to analyze this liquid‐solid interface and allows one to explain the log‐linear sensor response (similar to the Nernst equation used in general electrochemistry) of the FET measured by varying concentration of targets. At a fixed concentration, only the charges from the portion of the target molecule lying within a screening length are detected by the FET. Targets lying outside this length are screened by the ions in the electrolyte solution and not sensed.^[^ ^134^, ^135^, ^136^ ^]^ The screening length equals the thickness of the Stern layer and electrical double layer (the second layer is called the Debye length) formed by the ions in the electrolyte solution. As the screening length varies with the composition and the concentration of the electrolyte used in the FET,^[^ ^134^, ^136^ ^]^ ions in electrolyte critically define the sensitivity of the FET (in fact, any potentiometric) sensor. A recent study shows the reduction in the response of FETs (made with graphene channels) with the increase in the ionic concentration of the electrolyte for a wide range of targets (Figure 4d).^[^ ^135^ ^]^ The response can be improved by relaxing the screening effects when a heterodyne AC signal and a DC bias are applied to the FET to measure the response. The response can also be improved using a permeable polymer layer at the sensor surface that effectively increases the screening length.^[^ ^137^, ^138^ ^]^ This method allows measuring the response from the change in source/drain current monitored at a gate bias near the subthreshold region of the FET^[^ ^139^ ^]^ or using an inherently charged BRE that changes conformation, and thus the electrostatic environment closest to the electrode (APTA‐FET). The reduction of charge screening effects will make FETs more desirable for real‐time sensing of charged targets.

### 4.3 Quartz Crystal Microbalances and Cantilevers

Acoustic transducers generate signals in the form of frequencies. Measurable change in resonance frequency occurs depending on the mass changes due to binding events between receptors and target biomarkers. Frequency signals are fundamentally sensitive to target detection and provide real‐time and continuous measurements.^[^ ^140^ ^]^ Representative acoustic transducers are quartz crystal microbalance (QCM) and cantilever. A QCM (Figure 4e)^[^ ^141^ ^]^ is a generally used acoustic transducer with a standard structure and dimensions composed of a piezoelectric quartz crystal sandwiched between two gold electrodes. A QCM captures a mass variation in a specific area by detecting the changes in the frequency of a quartz crystal resonator.^[^ ^142^ ^]^ By immobilizing receptors onto the surface of the acoustic resonator, the resonance changes by the addition of a small mass due to biomarkers binding.^[^ ^143^ ^]^ QCMs have broad applicability to HHPM as they operate under vacuum as well as gas and liquid phases.^[^ ^143^ ^]^ Some examples of research progress include a twin QCM system integrated with flow injection analysis for the sequential detection of cortisol,^[^ ^144^ ^]^ an aptamer‐functionalized QCM for the detection of thrombin,^[^ ^145^ ^]^ and a peptide‐functionalized QCM for the detection of trypsin.^[^ ^146^ ^]^

The cantilever method can be used in both static and dynamic modes.^[^ ^147^ ^]^ Static mode is related to the electromechanical principle that uses deflection of the cantilever membrane due to a binding event on the cantilever surface. This deflection of the flexible cantilever beam is measured by piezomaterials located at the edge of the cantilever. Dynamic mode is related to acoustic measurement. In this mode, cantilevers act as harmonic oscillators, and resonance frequency changes are measured when additional biomarker molecules are attached to the oscillating cantilever. The cantilever (Figure 4f)^[^ ^148^, ^149^ ^]^ generally offers a more customized structure and reduced dimensions than QCM.^[^ ^150^ ^]^ A few review papers specifically dealt with cantilever biosensors, including micro/nano‐fabricated structures^[^ ^151^ ^]^ and dynamic modes.^[^ ^148^ ^]^

Whilst several cantilever‐based sensors have targeted biological and chemical analytes such as proteins and RNAs, not many researchers have focused on the detection of HHPB. One of the studies for HHPB utilized a conjugated polymer (oxaborole polymer + glycopolymer) as a sensing material to detect dopamine^[^ ^149^ ^]^ selectively. This polymer was deposited onto a cantilever surface to induce deflection change due to dopamine displacement on the polymer layer. While it achieved high sensitivity with a limit of detection of 5 × 10^−11^ mol L^−1^, the authors indicated that microcantilevers need to overcome challenges associated with response time and stability. Other study deposited a chitosan layer onto a cantilever surface to detect dopamine. By using an oxidizing process, dopamine's oxidation product was reacted with a chitosan sensing material resulting in cantilever bending with a tensile stress of 1.7 MPa.^[^ ^152^ ^]^ It also demonstrated chitosan's selectivity to dopamine by comparing it with ascorbic acid with little to no response. Some study utilized antibodies to detect the cardiac biomarkers creatine kinase and myoglobin.^[^ ^153^ ^]^ Cantilever surface stress was generated by antigen–antibody molecular interaction, resulting in a sensitivity of 20 µg mL^−1^. This work utilized an array‐type cantilever to detect multiple proteins and form a reference cantilever to eliminate noise factors such as thermal drifts and turbulences from undesired external events. While having favorable characteristics of continuous measurement and miniaturized size, cantilever‐based sensors still have challenges in practical aspects such as stability, durability, response time, and reproducibility. A recent study demonstrated a 3D printed microcantilever for biosensing applications (Figure 4g).^[^ ^154^ ^]^ This work suggested a one‐step printing strategy to fabricate cantilever arrays with intrinsic functionalization, showing promising results that can overcome reproducibility and fabrication complexity issues.

### 4.4 Enhanced Surface Plasmon Resonance

Plasmonically active metals (Ag, Au) respond to changes in the local surrounding environment (electronic, polarization, and refractive index) from binding events near the metal surface. These binding events come in the form of intense localized surface plasmon resonances (LSPR) for single metal nanostructures or surface plasmon resonances (SPR) in thin metallic films (Figure 4h).^[^ ^155^ ^]^ Additionally, plasmonic metals also participate in surface‐enhanced Raman scattering and plasmonic enhancement of fluorescence by exhibiting intense electromagnetic field enhancements that result in single molecule detection and amplification of fluorescence. LSPR measurements have been extensively used to determine binding affinities of antibody–antigen interactions with high accuracy, limits of detection, and as a basis for the fabrication of a variety of biosensors.^[^ ^156^ ^]^ However, their use in wearable sensors is much more limited in practice due in part to the sensitivity of optical properties of the environmental (temperature) and mechanical forces (stress, strain). More so, miniaturization requirements of components (spectrometer, light source) for integration with plasmonic sensing elements^[^ ^157^ ^]^ continue to provide issues for functional sensing platforms. Conceptually, LSPR measurements of single nanostructures overcome issues associated with mechanically induced optical changes from bending or stretching and, as a result, are more amenable to the development of wearable plasmonic biosensors. Notably, LSPR offers label‐free and real‐time measurements, low cost, high sensitivities, and compatibility with multiple fabrication methods and substrates.^[^ ^155^ ^]^ Recent advancements in the controlled synthesis of gold nanostructures have enabled tunable plasmonic properties, high monodispersity, and uniformity with respect to nanomaterial size, shape, and the ability to be scaled up to gram quantities.^[^ ^158^ ^]^ In all, these advancements in nanomaterial processing steps will lead to greater device reproducibility, increased manufacturability on a commercially viable scale, better performance and rapid development, and deployment of wearable plasmonic biosensors.

For the fabrication of wearable plasmonic biosensors, paper (cellulose), optical fibers (silica), and polydimethylsiloxane (PDMS)‐based microfluidics (lab‐on‐a‐chip) represent the most promising platforms for plasmonic devices and pertinent examples of current plasmonic sensing technologies. Paper substrates offer a simple, inexpensive, flexible, robust, and disposable platform to stabilize optically active gold nanostructures against aggregation for microfluidics and separation of chemical compounds. For example, the immobilization of gold nanomaterials on filter paper imparted stabilization to particles against acids, bases, salts, alcohols, heating, freezing, and organic solvents.^[^ ^159^ ^]^ To create plasmonic paper, gold nanostructures are adsorbed onto paper substrates with high nanoparticle loading by exploiting the simple adsorption and wicking processes of paper. Consequently, this produced a highly sensitive plasmonic paper device, when functionalized with BREs, to sense and detect biological targets.^[^ ^160^ ^]^ For example, the LSPR response of peptide‐functionalized gold nanorods immobilized on paper was utilized to detect low pm concentrations of human cardiac troponin.^[^ ^81^ ^]^ In addition to paper, dielectric waveguides in the form of optical silica fibers are ideally adapted for plasmonic measurements by directly coupling light propagation through an optical fiber to an LSPR active metal coating. In this optical configuration, the light source is integrated with an optical fiber and gold film to yield a responsive plasmonic sensor capable of interrogating binding interactions on the gold‐coated optical fiber tip by monitoring changes in the extinction spectra. As a result, optical plasmonic fibers have been effectively used as implantable sensors, medical diagnostics, and portable devices for environmental and food testing. Tilted fiber Bragg gratings imprinted on optical silica fibers were coated with a 50 nm gold film via sputter coating and functionalized with an antibody against a cancer antigen for SPR detection and diagnosis of cancer (Figure 4i).^[^ ^161^ ^]^ Additionally, fiber optic‐SPR sensors modified with borate polymers were used for the selective detection of glucose.^[^ ^162^ ^]^ Similar to paper and optical fibers, the integration of gold with PDMS has enabled the creation of microfluidic plasmonic chips for lab‐on‐chip applications. Generally, these platforms offer low sample volume, complex microfluidic designs, low cost, and high‐throughput manufacturing. For sensing, plasmonic‐based microfluidic sensors were created to detect bovine growth hormones using in situ grown gold nanoparticles functionalized with antibodies deposited within microfluidic channels.^[^ ^163^ ^]^ In this system, the plasmonic lab‐on‐chip device showed a sensitivity of 74 nm per RIU (refractive index units) and a detection limit of 185 × 10^−12^ m for growth hormones.

Collectively, these examples demonstrate the potential benefits of utilizing plasmonic materials on paper, optical fibers, and PDMS for wearable optical devices. By comparison to other nonoptical methods, the benefits of plasmonic sensors include lower limits of detection due to sensitivity of LSPR, ease of functionalization with BREs using gold surface chemistry, detection of a broad spectrum of biomarkers, ability to operate in complex biological fluids and simple integration on a variety of different substrates. The remaining challenges include 1) balancing the level of sensitivity with the distance between the optically active gold surface and the bound target imposed by the size of the BRE, 2) integration of plasmonic sensing platforms with off‐the‐shelf wearable components, and 3) needed improvements in miniaturization and packaging to accommodate a wearable and lightweight platform. In the near future, we envision the development and uses of alternative plasmonic materials beyond Au and Ag for wearable optical biosensors such as Al and Cu, bimetallic alloys and nanoparticle superlattices, reliance on machine learning for better plasmonic sensor design and performance, and continued miniaturization of components with an emphasis on micro‐spectrometers and smart phone compatibility.

### 4.5 Colorimetric Measurement

Colorimetric measurement detects a target biomarker through color changes observed by bare eyes or optical detectors for the quantitative measure. Generally‐used common colorimetric materials include color‐responsive dyes and metal nanoparticles that change their colors in response to target biomarkers. Color‐responsive dyes are usually used in either enzymatic sensors where the specific dyes interact with byproducts produced by enzyme binding events^[^ ^164^ ^]^ or photonic sensors where the chemically designed dyes interact with specific wavelength.^[^ ^165^ ^]^ The use of metal nanoparticles such as gold and silver nanoparticle is based on the plasmonic effect resulting in colorimetric shift.^[^ ^166^ ^]^ For example, gold nanoparticles with purple color in ionic solution change their color to red in the presence of a target molecule (dsDNA) due to the plasmonic absorption of the dispersed nanoparticles (Figure 4j).^[^ ^167^ ^]^

The Rogers group has intensively focused on colorimetric materials to detect biomarkers. The biomarkers were temporally resolved either through the use of microfluidic channels that progress clockwise around a patch with colorimetric sensors or through a continuous flow of sweat in a well containing saturated pH or chloride‐sensitive dye that can be used to measure flow rate analyte concentration.^[^ ^112^, ^168^, ^169^ ^]^ Enzymatic sensors can be created by relying on first‐generation principles, where the generation of hydrogen peroxide reacts with iodide in the presence of peroxidase to yield a color change from yellow to brown for glucose. The redox mediator NADH is consumed by formazans using diaphorase enzyme catalysis to mediate the generation of colorimetric changes (Figure 4k).^[^ ^164^ ^]^

The benefits of these methodologies are simplicity and the potential for a low cost. The drawback to this methodology is the need for the user to take a picture, thus reducing the ability to obtain real‐time data and the loss of time resolution from using larger pooled samples by definition averaged over time. For enzyme applications, the same limitations apply, as discussed in the above section on enzymatic sensors.

## 5 Practical Challenges on Wearable Human Heath and Performance Monitoring Sensors

Due to many physical, chemical, and biological variables in the operational setting, the biomarker sensors should have additional specific features to mitigate such environmental challenges. The features discussed below focus on wearing comfort, fluid manipulation, and enhanced sensor performances for stability/durability, continuous operation, and power management.

### 5.1 Skin‐Conformal Platforms

Flexible and stretchable substrates can be directly applied to the skin with conformal contact, which offers significant advantages over rigid substrates in the direct measurement of biomarkers from the skin surface. A number of representative research groups have demonstrated a variety of flexible and stretchable platforms noninvasively detecting biomarkers found on the skin.

**Figure 5:**
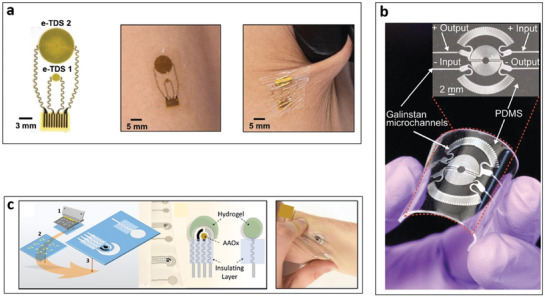
Flexible substrate‐based biomarker sensing platforms. a) Epidermal electronic systems to measure thermal properties of human skin. Reproduced with permission.^[^ ^173^ ^]^ Copyright 2018, Wiley‐VCH. b) Physical biomarker sensors targeting pressure related to human skin by microfluidics‐based diaphragm pressure sensor. Reproduced with permission.^[^ ^187^ ^]^ Copyright 2017, Wiley‐VCH. c) Tattoo‐type sensors utilizing a sweat‐based epidermal enzymatic biosensor for the assessment of Vitamin C levels. Reproduced with permission.^[^ ^196^ ^]^ Copyright 2020, American Chemical Society.

One of the research groups has demonstrated nanoscale ultrathin silicon substrates offering flexible mechanical characteristics to the semiconductor channel.^[^ ^170^ ^]^ Nanometer‐thick flexible semiconductor substrates, along with flexible materials such as polyimide (PI), ecoflex, and polyethylene terephthalate (PET), the group has shown various wearable skin sensors reporting physical biomarkers including skin temperature^[^ ^170^, ^171^, ^172^, ^173^ ^]^ skin impedance (hydration),^[^ ^174^, ^175^, ^176^ ^]^ sweat rate,^[^ ^177^, ^178^, ^179^ ^]^ blood flow,^[^ ^180^, ^181^ ^]^ skin mechanical property,^[^ ^176^, ^182^ ^]^ and cardiogram.^[^ ^51^ ^]^ An epidermal electronic system was built to measure the thermal properties of human skin (**Figure** **5**a).^[^ ^173^ ^]^ This epidermal system utilized an ecoflex silicone layer as a substrate on which PI insulation layers sandwiched gold electrodes. The 100 µm thin structure enabled a skin‐conformal thermometer capable of monitoring the blood flow and hydration status.

Another group has utilized flexible substrates such as PET, PDMS, and polyetherimide to build wearable sweat sensors detecting electrochemical biomarkers including Ca^2+^,^[^ ^67^ ^]^ pH,^[^ ^67^ ^]^ glucose,^[^ ^183^, ^184^ ^]^ Na^+^,^[^ ^185^ ^]^ K^+^.^[^ ^186^ ^]^ While focusing on sweat compositions, the group recently worked on tactile sensors targeting physical biomarkers on human skin (Figure 5b).^[^ ^187^ ^]^ Microfluidics‐based diaphragm pressure sensors with a Wheatstone bridge design enabled measuring low ranges of pressure stimulation from pulse waves and tactile forces. To realize the detection limit of sub‐100 Pa pressure, a Galinstan‐filled 70 µm microchannel was utilized to form diaphragm‐type pressure sensors. The tactile sensor was used to report heart rate and haptic information as gloves or wristband‐type platforms.

Tattoo‐type skin sensors^[^ ^188^ ^]^ have been developed by another group, where temporary transfer tattoo‐based paper is used as the main substrate. The group has shown tattoo‐like sensors detecting Na^+^,^[^ ^189^ ^]^ glucose,^[^ ^190^, ^191^, ^192^, ^193^ ^]^ and lactate^[^ ^194^, ^195^ ^]^ in sweat and saliva. One of their more recent work showed tattoo‐type sensors utilizing a sweat‐based epidermal enzymatic biosensor to assess Vitamin C levels (Figure 5c) on the skin.^[^ ^196^ ^]^ An immobilized ascorbate oxidase on the tattoo‐based flexible substrate was used to electrochemically monitor time‐dependent nutrition level from the saliva or tear samples.

While skin‐conformal sensors have emerged as promising wearable platforms, fabrication faces challenges due to the time‐consuming and complicated procedures with low yield rates and poor reproducibility. Recent flexible skin biomarker sensors started widely adapting advanced fabrication techniques such as roll‐to‐roll printing, screen printing, and 2D inkjet printing techniques, which significantly simplify and expedite the fabrication process without conventional MEMS cleanroom facilities. One example of such printing techniques utilized roll‐to‐roll gravure printed electrodes with a minimum width of 0.5 mm, which potentially enabled mass fabrication with up to 150 m PET substrate roll and simplified the fabrication procedures^[^ ^197^ ^]^ while producing high‐quality sensor units. The demonstrated electrochemical sensors fabricated by this method also measured potassium, sodium, and glucose. We anticipate that such advanced fabrication techniques will play significant roles.

### 5.2 Body Fluidics

Sample collection is a critical step in any real‐time analysis. However, most noninvasive biofluids are not typically produced or extracted at sufficiently high rates. While the human body typically produces between 0.5 and 1.5 L of saliva per day and thus continuously provides a moist environment for embedding a sensor,^[^ ^198^ ^]^ sweat and ISF extraction produce additional problems that have been addressed using multiple methods. The problem with a sampling of sweat is that sweat rate varies drastically, even within an individual, depending on hydration level, heat stress, and exertion. Under many conditions, the sweat rate will be too low to enable noninvasive sampling. Therefore, artificial stimulation of sweat has been explored using small molecules (e.g., carbachol^[^ ^199^ ^]^ and pilocarpine^[^ ^200^, ^201^, ^202^, ^203^ ^]^) that stimulate either the muscarinic or nicotinic receptors within the sweat gland. These drugs are delivered into the sweat gland to stimulate sweating using iontophoresis, typically performed for sweat rate tests that enable the detection of cystic fibrosis. Pilocarpine is known to have a relatively short half‐life compared to carbachol, a homolog to acetylcholine that uses a carbamate instead of an ester functional group, prolonging half‐life by preventing hydrolysis by acetylcholine esterase; This results in a sweat response that lasts on the order of hours instead of minutes.^[^ ^204^ ^]^ Few research works have demonstrated the analyte changes upon stimulated sweat secretion versus natural sweat secretion and which (if either) serves as a better biofluid for monitoring biomarker changes. In theory, both simulated and natural sweats should have the same analyte ratios, as it is hypothesized that analyte secretion into the sweat gland occurs through a sweat rate‐independent pathway. The secretion of molecules into the sweat is constant, and therefore as sweat rate increases, the concentration in the sweat decreases.^[^ ^205^ ^]^ Therefore, analyte concentration and their relative ratios may be used in lieu of sweat rate measurements to correlate biomarker expression to physiological states.

**Figure 6:**
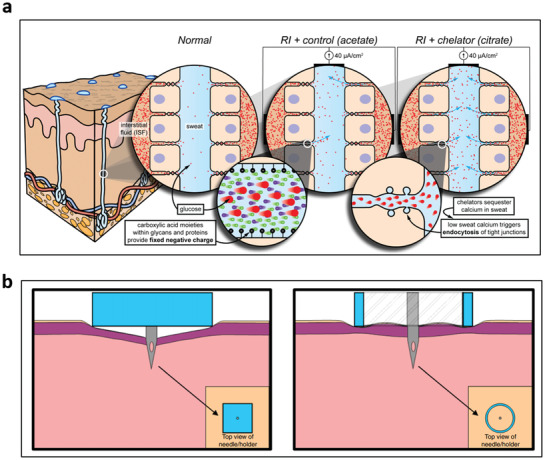
Body fluid manipulation techniques. a) Iontophoretic delivery of a chelating agent for breaking apart the tight junctions through sequestration of calcium, increasing the flux of analytes in conjunction with reverse iontophoresis. Reproduced with permission.^[^ ^205^ ^]^ Copyright 2018, PLoS. b) Hollow needles with substantially greater extraction of ISF due to the inherent flow restrictions with any polymer‐based methodology. Reproduced with permission.^[^ ^20^ ^]^ Copyright 2018, Springer Nature.

Iontophoresis has been applied previously to enhance analyte flux from the analyte‐rich interstitial fluid to sweat, most notably the Glucowatch,^[^ ^206^ ^]^ to measure flux and link it to physiological events. In short, the flow of sodium ions toward the anode coating the negatively charged surface of the cellular membranes produces an ionophoretic field that causes nonpolar molecules to migrate toward the electrochemical cathode. However, the flux is relatively small, consistent with the extracellular matrix of cells, providing a high resistance path to extract analytes of interest. Recent efforts on miniaturizing iontophoresis devices to a tattoo made them easier to implement as wearable sensors.^[^ ^76^ ^]^ Improvements are still needed to further increase the analyte flux to levels close to those found in interstitial fluid. Iontophoretic delivery of a chelating agent could break apart the tight junctions through sequestration of calcium, substantially increasing the flux of analytes in conjunction with reverse iontophoresis (**Figure** **6**a).^[^ ^205^ ^]^ A similar approach has been used previously in gut epithelium to improve drug delivery,^[^ ^207^ ^]^ but never for analyte extraction. More systematic work remains to obtain controlled parameters or understand the link between analyte concentration in sweat and iontophoretic current. Nonetheless, the ability to enrich sweat using a combination approach is highly promising and shows great potential in bridging the gap between sensor performance and analyte concentration.

ISF provides a compromise between invasive biofluids such as blood and noninvasive biofluids such as sweat. ISF requires piercing of the skin, typically via microneedles, into the dermis, through the epidermis. However, the shallowness of the needles prevents the piercing of capillaries and, thus, the extraction of blood. Moreover, the risk of blood‐borne pathogens in the resulting regions bypassing the intrinsic defenses of the skin barrier (proteases, antimicrobial peptides, and tight junctions^[^ ^208^ ^]^) remains a critical challenge. ISF must be extracted, allowing for the control of flow rates to the device. Often, this relies on wicking principles to overcome the ISFs viscosity, especially in the fibrous extracellular matrix created by the collagen matrix. Both hollow needles^[^ ^20^ ^]^ and hydrogels have been explored for fluid extraction. The use of hollow needles has demonstrated substantially greater extraction of ISF, hypothesized to be due to the inherent flow restrictions with any polymer‐based methodology (Figure 6b).^[^ ^20^ ^]^ Additionally, hollow needles are presumed to be free of analyte filtration effects that may be problematic with any polymeric matrix. Osmotic pumping has been demonstrated to increase extraction rates that can be simulated by applying a vacuum or other pressure‐based forces.^[^ ^209^ ^]^ However, the difficulty of integrating these recent findings into wearable/portable real‐time sensors has prevented their deployment. To date, the sensor is embedded as part of the microneedle (most typically for enzymatic^[^ ^210^ ^]^ and potentiometric^[^ ^211^ ^]^ sensors), and perfusion is used to pump and extract the ISF^[^ ^210^ ^]^ actively.

### 5.3 Miniaturization Strategies

Aside from sampling and sensing of biomarkers, integration into wearable platforms poses a significant challenge for sensor development, particularly when packaging into the suitable form factor. More device per unit area of skin demands smaller electronics, patches, interconnects, communication, and power systems. Recent progress in nano/microfabrication techniques has dramatically enabled the miniaturization of such systems suitable for portable/wearable sensor platforms.^[^ ^212^ ^]^ It has been essential to integrate multiple biomarker sensors, and thus the design of an efficient device structure has been emphasized to maintain the total platform size. One example is to design multilayers or efficient form factors to integrate multiple sensors to increase space efficiency and reduce sensing area.

**Figure 7:**
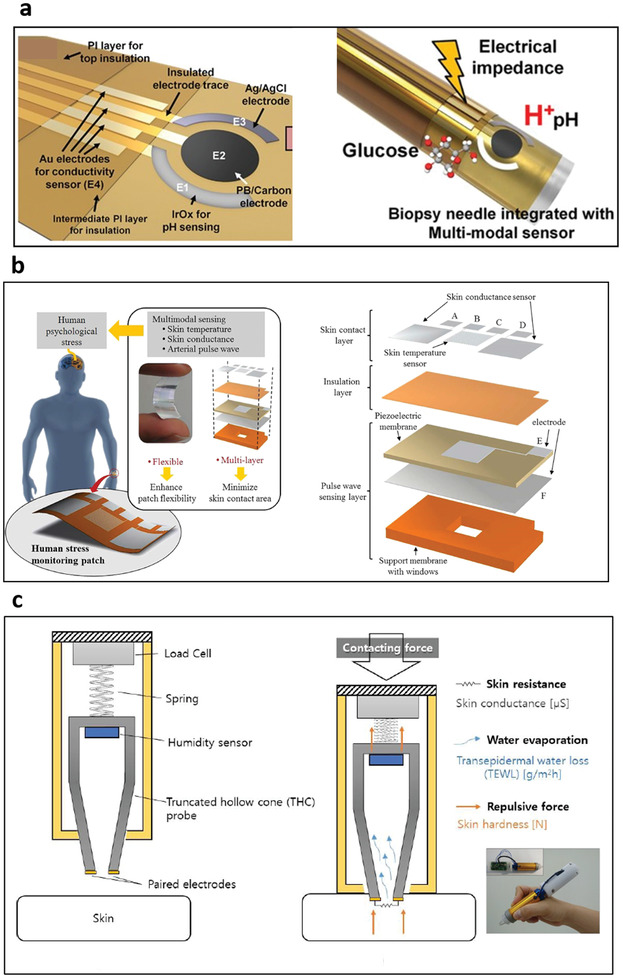
Integration designs of multimodal platforms for reducing measuring site. a) Physical/chemical sensor arrays where electrodes for a conductivity sensor were patterned in the middle of each electrode for glucose and pH sensing applications, showing the fabrication of the multimodal sensors placed on a surface of the small diameter needle. Reproduced with permission.^[^ ^213^ ^]^ Copyright 2020, Elsevier. b) Reduced size of stress monitoring patch with a size comparable to that of a stamp, while measuring multiple physical biomarkers such as skin temperature, skin conductance and pulse wave. Reproduced with permission.^[^ ^214^ ^]^ Copyright 2016, Springer Nature. c) Specific design of a probe that enabled three different biomarker measurements where the specially designed truncated hollow cone probe efficiently contained a force sensor above a conductance sensor with a sweat rate sensor placed inside the hollow probe. Reproduced with permission.^[^ ^215^ ^]^ Copyright 2019, MDPI.

A few research works deployed this specific multilayer structure to realize multimodality without size increment. For example, physical/chemical sensor arrays demonstrated a capability of measuring three biomarkers on a biopsy needle (**Figure** **7**a).^[^ ^213^ ^]^ Electrodes for a conductivity sensor were patterned in the middle of each electrode for glucose and pH sensing applications, achieving the fabrication of the multimodal sensors placed on the surface of the small‐diameter needle. This multi‐insulation layer‐based design allowed the downsizing the sensing platform to fit the needle. A recent work demonstrated the fabrication of a stress monitoring patch with a size comparable to that of a stamp (25 mm × 15 mm × 72 µm) while measuring multiple physical biomarkers such as skin temperature, skin conductance, and pulse wave (Figure 7b).^[^ ^214^ ^]^ This monitoring patch utilized multi‐layer integration on the pulse wave sensor by using a polyimide substrate with windows to make strategic stacking of add‐ons (i.e., temperature and conductance sensors), resulting in a multimodal patch with high flexibility. Meanwhile, the design of a specific form factor has also been deployed to optimize multimodality. One of the recent studies demonstrated a skin probe that enabled three different biomarker measurements (Figure 7c).^[^ ^215^ ^]^ The skin probe included a truncated hollow cone probe^[^ ^216^ ^]^ housing a force sensor, a thermometer, and a conductance sensor with the capability to measure a sweat rate. The narrow end and closed hollow structure characteristics enabled adequate skin hardness and sweat rate detections, enabling one to measure three different physical biomarkers simultaneously.

The above research progress implies that multilayer structure and form factor‐efficient design integrated with multiple biomarker sensors are promising strategies to achieve miniaturized wearables conforming to the body for maximum user comfort.

### 5.4 Environmental Stability

**Figure 8:**
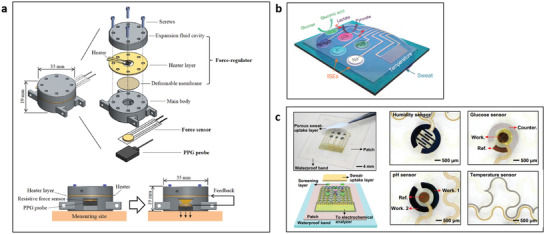
The use of add‐ons for improving accuracy and stability. a) Integration of a force regulator with a PPG sensor contact force between the skin and a PPG sensor constantly for the consistent and stable PPG measurement results independent of body movements. Reproduced with permission.^[^ ^277^ ^]^ Copyright 2018, AIP Publishing. b) Integration of a micro‐sized RTD type temperature sensor next to several sensing modules to compensate measurement results depending on the temperature. Reproduced with permission.^[^ ^75^ ^]^ Copyright 2016, Springer Nature. c) Integration of temperature, humidity and pH sensors to obtain more accurate glucose measurement results where the three additional elements were monolithically integrated with the glucose‐sensing element on the flexible substrate. Reproduced with permission.^[^ ^68^ ^]^ Copyright 2017, AAAS.

Biomarker sensors for either wearable or on‐site detection in the operational setting commonly face inconsistent environmental/chemical exposures, unlike controlled laboratory settings. Moreover, the human body adds movement as an additional physical interference factor that affects the stability of the wearable sensor. In particular, physical biomarkers such as pulse waves and blood flow are heavily valuable to body movement. For example, contact force variation causes more than 70% differences in measurement results for the pulse wave measurement.^[^ ^217^ ^]^ While conventional methods address this issue by applying a constant pressure using a spring clip, it commonly fails to prevent measurement variations from body movement‐induced contact force changes. Integration of the force sensor into pulse wave or blood flow sensors was utilized to compensate the measurement depending on the level of the force measured in parallel with pulse wave or blood flow measurement.^[^ ^218^ ^]^ However, adding force sensor does not fundamentally solve the contact force effect since the compensation coefficient differs from person to person and site to site. The integration of a force regulator with a PPG sensor (**Figure** **8**a)^[^ ^277^ ^]^ was demonstrated to solve this issue. The integrated force regulator keeps a constant contact force between skin and a PPG sensor, realizing consistent and stable PPG measurement results independent of body movements.

Biochemical sensors that utilize binding receptors rely on the physicochemical reactions and are thus critically affected by surrounding environmental conditions such as temperature, humidity, ions, and pH. In order to obtain accurate detections under such environmental variations, several research groups have integrated additional sensing elements that report the ambiance. One study, for example, implemented a temperature sensor next to the sensing modules (Figure 8b).^[^ ^75^ ^]^ Micro‐RTD as temperature sensors calibrated responses from metabolites and electrolytes and measured skin temperature to understand human physiological states. Other study implemented not only temperature sensors but also humidity and pH sensors to obtain more accurate glucose measurement results (Figure 8c).^[^ ^68^ ^]^ The three environmental sensors were monolithically integrated with the glucose monitor on the flexible substrate, where the temperature and humidity levels were measured using RTD and impedance methods, respectively. The pH sensor utilized a polyaniline‐deposited working electrode and a Ag/AgCl electrode to measure the open‐circuit potential between the two electrodes. The results indicated that pH‐based compensation processes enhance accuracy in the glucose level measurement.

Nano/microfabrication technology has been enabling the integration of additional complementary sensors into biomarker sensor platforms. Further studies are expected to build reliable calibration curves for compensation processes from the complex data obtained from such multiplexed monolithic sensing systems.

### 5.5 Sensor Recovery

**Figure 9:**
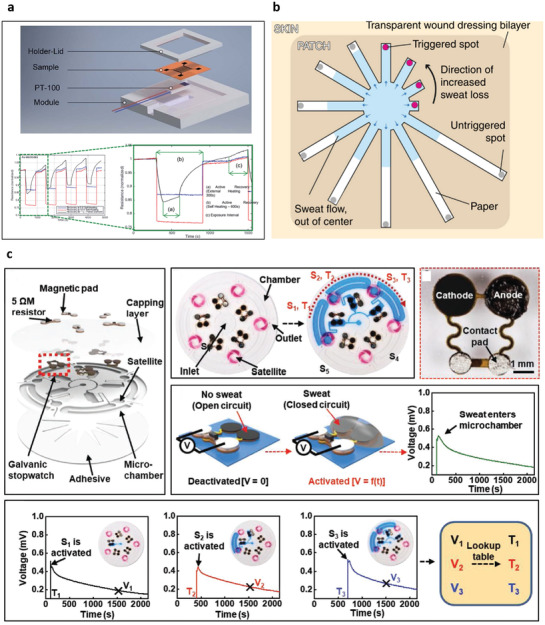
The strategies for improving sensor recovery time and continuous measurement. a) Self‐recovering gas sensors by using CNTs as both the sensing element and heater, significantly improving baseline drift and response time. Reproduced with permission.^[^ ^221^ ^]^ Copyright 2018, Royal Society of Chemistry. b) Paper‐based microfluidic skin patches where channels are patterned and radially arranged with different lengths to realize the discrete real‐time measurement of sweat secretion. Reproduced with permission.^[^ ^226^ ^]^ Copyright 2019, Springer Nature. c) Sweat collection through microchannels where the passive valves were used for chronometric collection of sweat, resulting in time‐dynamic analysis of sweat analysis using galvanic stopwatches. Reproduced with permission.^[^ ^227^ ^]^ Copyright 2019, Wiley‐VCH.

Chronological sensor data with proper intervals allows the users to prepare appropriate diagnostic and mitigation measures. In order to enable continuous biomarker measurements, the degree and rate of sensor recovery need to be accounted. In particular, biochemical and chemical sensors have preferred strong affinity receptors due to their high sensitivity and selectivity. However, high‐affinity receptors can also be the source of irreversibility on the operating sensor, thus prohibiting continuous sensing once detection events occur. Several methods have been adapted to improve the recovery processes of biomarker sensors. For example, carbon nanotube (CNT) gas sensors showing a strong binding affinity to NO_2_ and taking longer than 12 h recovery time^[^ ^219^ ^]^ in ambient temperature were applied with either increased temperature or UV light^[^ ^220^ ^]^ to facilitate the desorption. In addition, self‐recovering gas sensors was demonstrated, where CNTs served as both the sensing element and heater (**Figure** **9**a).^[^ ^221^ ^]^ By applying a voltage to the CNT sensing element, recovery processes significantly improved in terms of baseline drift and response time compared to applying an external temperature of 80 °C. Such self‐heating features have been used in many gas sensing applications^[^ ^222^, ^223^ ^]^ due to their benefit to the faster recovery time and power management.

Contrary to the chemical/biochemical sensors, most sensors tailored to physical biomarkers such as temperature and pressure tend to show fast response time and recovery time, reporting near real‐time chronological data. However, certain physical biomarkers, such as sweat rate, inherently encounter long sensor recovery time. For example, sweat rate measurements using a closed chamber consist of a humidity sensor placed inside a chamber and measures humidity change to derive sweat rate. Since the humidity chamber requires a ventilation process to refresh the humidity level, the closed chamber method typically shows inconsistency in continuous sweat rate measurements. In order to mitigate such ventilation issues, a sweat rate measurement module was integrated with a thermopneumatic actuator.^[^ ^224^, ^225^ ^]^ The actuator lifts the humidity chamber away from the skin, enabling continuous measurements by enhancing the ventilation. Recently, microfluidics has been rising as a skin‐attachable sweat rate sensor. The microfluidics are cost‐effective and compatible with several biochemical sensing elements. Microfluidics‐based sweat rate sensors often use capillary force, enabling quantitative detection of the amount of water generated from the skin by monitoring the length of the channel sweat passed through. One of the recent microfluidics‐based sweat rate sensors introduced paper‐based microfluidic skin patches where channels are patterned and radially arranged with different lengths to realize the discrete real‐time measurement of sweat secretion (Figure 9b).^[^ ^226^ ^]^ Other study presented sweat collection through microchannels where the passive valves were used for chronometric collection of sweat, resulting in time‐dynamic analysis of sweat analysis using galvanic stopwatches (Figure 9c).^[^ ^227^ ^]^ This microfluidics‐based stopwatch sensor integrated chloride and pH sensing elements for the semicontinuous measurement of multiple biomarkers.

### 5.6 Power

Small commercial batteries such as coins or lithium batteries have been commonly applied to power a sensor. Such batteries tend to be relatively heavy and bulky for skin‐conformal wearable applications and show minimal power capacities to operate sensors for a prolonged period of time (about 24 h). Advanced power generation, storage, and management technologies have emerged to solve these power issues, and some well‐organized review papers^[^ ^228^, ^229^, ^230^, ^231^ ^]^ have already been published. Here, we show a few most recent advancements (since 2018) that have successfully integrated power sources with biomarker sensors and exhibiting wireless operation.

**Figure 10:**
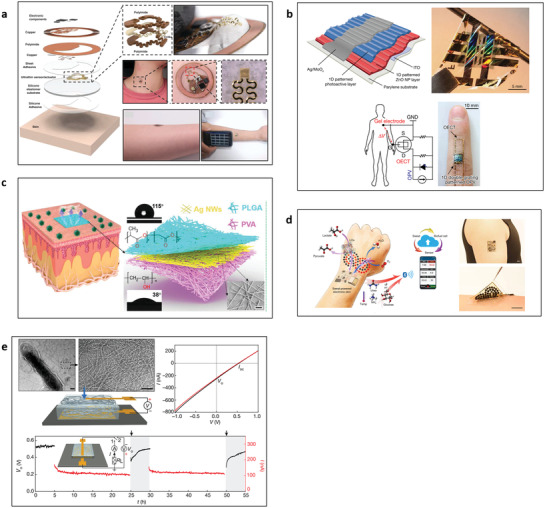
Wireless power and energy harvesting for biosensors. a) Near field communication (NFC) as a power supply system that was integrated with the skins thermal property sensing element. Reproduced with permission.^[^ ^232^ ^]^ Copyright 2018, Wiley‐VCH. b) Self‐powered conformal skin sensors based on the flexible photovoltaics where an organophotovaltaic (OPV) power source was integrated with a sensing element in the form of an electrochemical transistor to measure cardiac signals from human skin. Reproduced with permission.^[^ ^234^ ^]^ Copyright 2018, Springer Nature. c) Electronic skin based on a nanofiber triboelectric nanogenerator (TENG) that serves as an energy harvester converting mechanical energy into electricity based on the coupling effect of contact electrification and electrostatic induction. Reproduced with permission.^[^ ^235^ ^]^ Copyright 2020, AAAS. d) Battery‐free, perspiration‐powered electronic skin (PPES) that harvests energy from human sweat by using lactate biofuel cells (BFCs). Reproduced with permission.^[^ ^236^ ^]^ Copyright 2020, AAAS. e) Protein nanowire‐based power generation from ambient humidity. Reproduced with permission.^[^ ^237^ ^]^ Copyright 2020, Springer Nature.

Near‐field communication (NFC) served as a power supply system that was integrated with the skin temperature sensor (**Figure** **10**a).^[^ ^232^ ^]^ The NFC device consisted of inductive coils on a flexible PCB board enabling wireless power harvesting and other electronic components for NFC data transmission. This method allowed the thermal actuator and sensor to be wirelessly operated by a nearby smartphone or radio‐frequency reader without using a tangible power source upon the platform. Another example utilizing NFC technology was a neonatal monitoring device.^[^ ^233^ ^]^ Here, all sensor elements were placed upon infant skin surfaces and wirelessly powered by the external sources placed underneath the crib. This sensing mode allows for improved efficiency and comfort for both infants and guardians.

Flexible photovoltaics served as self‐powered conformal skin sensors.^[^ ^234^ ^]^ An organophotovoltaic (OPV) power source was integrated with a sensing element in the form of an electrochemical transistor to measure cardiac signals from human skin (Figure 10b).^[^ ^234^ ^]^ A flexible OPV element was composed of a specific photoactive layer and a layer of zinc oxide nanoparticles (ZnO NPs). This structure served as the electron‐transporting platform demonstrating a good power‐conversion efficiency of 10.49% and power‐per‐weight of 11.46 W g^−1^ (an adequate amount of power for mobile device applications). This self‐powered conformal sensor integrated with a cardiac sensor successfully exhibited high performance (40.02 decibels of signal‐to‐noise ratio) of human physiological signals.

Electronic skin based on a nanofiber triboelectric nanogenerator (TENG) was demonstrated. TENG served as an energy harvester converting mechanical energy into electricity based on the coupling effect of contact electrification and electrostatic induction (Figure 10c).^[^ ^235^ ^]^ TENG consisted of the silver nanowire electrode sandwiched between a polylactic‐*co*‐glycolic acid layer on the top and a triboelectric layer of polyvinyl alcohol as the bottom substrate surface. The TENG‐based e‐skin exhibited a maximum peak power density of 0.013 mW cm^−2^ and pressure sensitivity of 0.011 kPa^−1^, realizing the self‐powered monitoring of physiological signal (e.g., pulse wave and respiration) and joint movement.

Lactate biofuel cells (BFCs) demonstrated a battery‐free, perspiration‐powered electronic skin (PPES) that harvests energy from human sweat (Figure 10d).^[^ ^236^ ^]^ Consisting of 0D to 3D nanomaterials, BFCs enabled continuous monitoring of key metabolic biomarkers and wirelessly transmitted the personalized information to a user interface via Bluetooth low energy.^[^ ^236^ ^]^ The PPES exhibited a power density of 3.5 mW cm^−2^ for lactate BFCs cells in human sweat and a 60 h continuous operation. It selectively monitored key metabolic analytes (e.g., urea, NH_4_^+^, glucose, and pH) and skin temperature during prolonged physical activities and wireless transmission of data to the user interface using Bluetooth.

A protein nanowire demonstrated power generation obtained from ambient humidity (Figure 10e).^[^ ^237^ ^]^ The thin film was fabricated based on the conductive protein nanowire from the bacteria called Geobacter. A protein nanowire film with a thickness of 7 µm could provide about 8.5 mW cm^−2^. The power generation mechanism used an ambient water molecule as a charge carrier based on the humidity gradient inside the film to provide voltage output. This nanowire device was able to operate LED and LCD panels by stacking several films to increase power generation.

Along with the above studies introduced so far, piezoelectric^[^ ^238^, ^239^ ^]^ and thermoelectric^[^ ^240^, ^241^ ^]^ have also been widely investigated to self‐power biosensors. Although most of the energy harvesting research revealed is not capable of harvesting large amounts of power density for real‐time detection (e.g., 30 Hz sampling), near real‐time discrete detection (e.g., periodic sampling for every 2 min) of biomarkers seems to be a real possibility for future and current technologies.

### 5.7 Commercial Products

Recently, the market for wearables has been immense and expanding dramatically. For example, worldwide spending on wearable devices was ≈$81.5 billion in 2021, an 18.1% increase from $69 billion in 2020, according to the report from Gartner, Inc. (www.gartner.com). The most widely commercialized wearable sensors are watch‐type platforms such as Apple Watch (Apple Inc., Cupertino, CA, USA) and Galaxy Watch (Samsung Electronics, Suwon, South Korea). The watches monitor and report health or activity status primarily by sensing physical traits such as heart rates and blood oxygen level, using optical emitters and detectors. While these optical devices provide rapid and abundant physical data, they suffer significant inaccuracy or inability to report higher HHPM other than the traits mentioned above. Both the proliferating market of the established wearables and their lack of precision make the demand for highly accurate and physiologically/psychologically relevant HHPM sensors extremely high.

One of the most established commercially developed wearable sensors has been focused on glucose monitoring in body fluids.^[^ ^242^ ^]^ Wearable glucose sensors initially started with the blood‐based optical or thermal detection methods with a form factor of a clip such as Gluco Track (Integrity Applications, Or Yehuda, Israel) or a waistband such as Optical Glucose Meter (C8 MediSensors, Inc., San Jose, CA, USA). However, such platforms rely on indirect detection of blood composition underneath the skin layer, and thus glucose estimation accuracy could be low compared to the direct blood sampling method. For better access to body fluid, minimally invasive ISF‐based glucose wearable sensors have developed a patch form factor such as Freestyle Libre (Abbott, Chicago, IL, USA) and CGM (Nurisense, Chicago, IL, USA). In addition, for direct access to body fluid, some wearable sensors focused on tears using a contact lens such as Google Smart Contact Lense (Google, Moutain View, CA, USA). This platform used a miniaturized wireless chip and sensor embedded in the contact to measure glucose levels constantly. However, one of the critical challenges for tear‐based biosensor platforms was the weak correlation between the concentration of metabolites in biofluid and blood. Therefore, a fundamental study on in‐depth biomarker analysis to probe the correlation between biomarkers and HHP is crucial to realizing reliable and consistent monitoring capability. More recently, wearable sweat‐based biomarker sensors have emerged. For example, Gx Sweat Patch (Epicore Biosystems, Cambridge, MA, USA) is a sweat‐based patch to monitor athletes' hydration and exercise levels. This wearable patch uses microfluidic technology and colorimetric detection, captures sweat throughout the exercise, and measures sweat rate and sodium composition, showing cost‐effective and practical sweat sample collection features. We anticipate that more commercial platforms will be on the market by expanding the biomarkers and achieving practical solution strategies for continuous real‐time monitoring.

## 6 Conclusions

Here, a broad range of biosensor technologies that should be accounted for in the HHPM applications were reviewed. Moreover, this paper addressed the HHPBs found in body fluid either minimally or noninvasively, in breath, or on the skin to provide perspectives in the HHPB characteristics such as concentrations/measurands ranges, HHP relevance, and pros/cons. We looked into biomarker sensing principles using ionophores, BREs, and other direct detection methods depending on the types of HHPBs. We then addressed the widely used transducers including EIS/SWV, FET, QCM/cantilever, SPR, and colorimetric method to understand their form factors and working principles transducing BRE activity into measurable signals. Lastly, we identified practical challenges and mitigation strategies by discussing skin‐conformal platforms, body fluidics, miniaturization strategies, environmental stability, sensor recovery, power, and commercial products.

While recent progress on biosensor technologies is noticeable toward enabling HHPM, it is crucial to appropriately leverage such technologies to realize successful monitoring systems. This review paper presents 1) multimodality and 2) reconfigureable modules, as the prospects and the future development trends in biomarker sensing.

Multimodality is to leverage multiple biomarkers to complement the pros/cons of each type of biomarkers for highly reliable HHPM. For example, we could carefully choose the list of biomarkers optimized for the specific area of interest on HHPM. For example, the biochemical biomarkers such as ions, metabolites, and neurotransmitters are likely to offer in‐depth insights and accurate estimations on human status. However, their measurement is relatively slow and errors from fouling, nonspecific binding, and chemical degradation. On the other hand, physical biomarkers, such as pulse waves and skin impedance, offer straightforward measurements and immediate responses with near real‐time detection. However, these only provide systemic responses and not the underlying biological processes that fully report on human status. Therefore, a future development trend would be building multimodal biosensors capable of detecting both biochemical and physical biomarkers, such as chemical‐electrophysiological hybrid biosensors^[^ ^243^ ^]^ (measuring lactate and electrocardiograms) and microfluidic sweat analysis patches^[^ ^186^ ^]^ (measuring ions, metabolites, and sweat rate).

**Figure 11:**
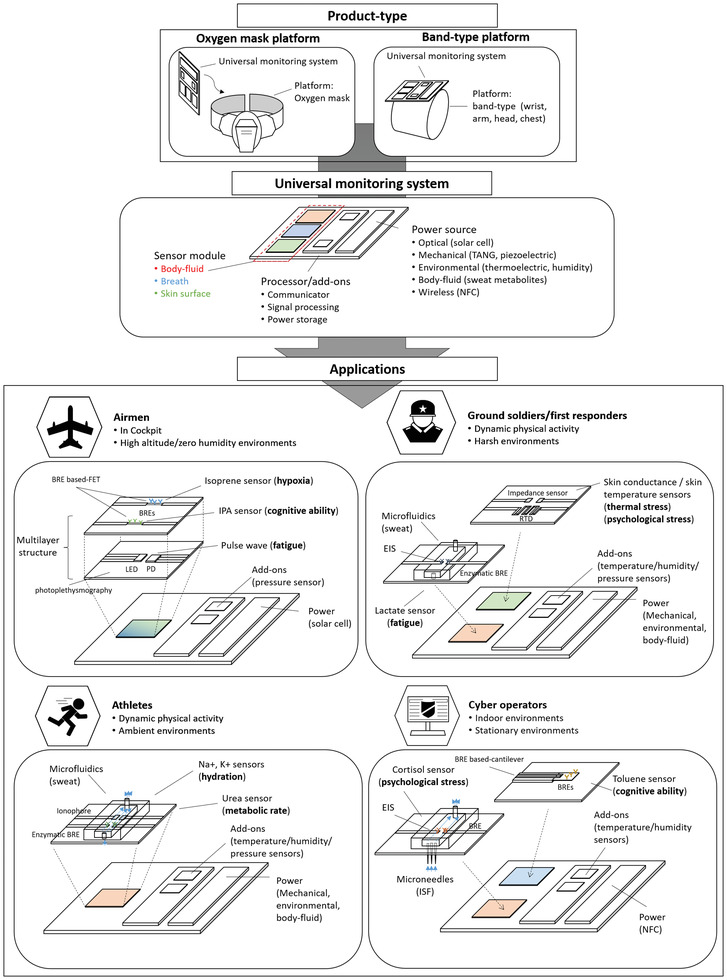
Product strategy for reconfigurable and cost‐effective manufacturing via module elements. The product type is divided into an oxygen mask platform and a band‐type platform, where a universal monitoring system is equipped. The universal monitoring system consists of sensor modules (body fluid‐, breath‐, or skin surface‐based), processor/add‐ons, and power source. Each module element can be customized to be inserted into the universal monitoring system, depending on the target applications (airmen, ground soldiers/first responders, athletes, and cyber operators) and their environmental conditions.

Reconfigurable modules provide various modular, interchangeable, and compatible components for meeting the wide range of end‐user demands in commercializing wearable HPPM. **Figure** **11** shows a schematic illustration of such modular biomarker sensing platforms. Here, the HHPM product consists of sensor modules (body fluid, breath, or skin surface‐based sensors), processors/add‐ons, and power sources. The universal monitoring system is amendable to adding/removing/replacing with proper sensor modules matching to the operational environments and the targets of interest. Also, appropriate add‐ons (e.g., pressure sensor) and power modules (e.g., solar cell) can be added to the specific operational environments. This modular product could be future trends to offer cost‐effective manufacturing for product transition by utilizing portable and tractable module elements. To further show a comprehensive concept of this sensor module strategy, we show various examples of sensor modules suitable for several applications (i.e., airmen, first responders, athletes, and cyber operators). In this regard, it is important to broadly understand biosensor technologies to realize such leveraged product platforms.

In conclusion, despite the technical challenges, the advances in miniaturized electronics, material science, and human biology/physiology, along with the progress in the multitude of advanced sensor approaches tailored for the biomarkers of interest, the scientific community is rapidly moving closer to the ultimate goal of real‐time HHPM.

## Conflict of Interest

The authors declare no conflict of interest.

## References

[1] M. C. Brothers , M. Debrosse , C. C. Grigsby , R. R. Naik , S. M. Hussain , J. Heikenfeld , S. S. Kim , Acc. Chem. Res. 2019, 52, 297. 30688433 10.1021/acs.accounts.8b00555

[2] J. Kim , A. S. Campbell , B. E. F. de Ávila , J. Wang , Nat. Biotechnol. 2019, 37, 389. 30804534 10.1038/s41587-019-0045-y PMC8183422

[3] M. Brothers , D. Sim , A. Islam , J. Slocik , B. Maruyama , M. Rubenstein , R. Naik , S. Kim , ECS Meet. Abstr. 2019, 28, 1370.

[4] W. Tharion , W. J. Tharion , A. W. Potter , C. M. Duhamel , A. J. Karis , M. J. Buller , R. W. Hoyt , J. Sport Hum. Perform. 2013, 1, 14.

[5] M. J. Buller , W. J. Tharion , C. M. Duhamel , M. Yokota , Ergonomics 2015, 58, 1830. 25967760 10.1080/00140139.2015.1036792

[6] D. Manzey , B. Lorenz , V. Poljakov , Ergonomics 1998, 41, 537. 9557591 10.1080/001401398186991

[7] R. W. Clemente Fuentes , C. Chung , Military, Civil and International Regulations To Decrease Human Factor Errors In Aviation, StatPearls Publishing, Treasure Island, FL 2020. 31536244

[8] G. Li , D. Wen , J. Mater. Chem. B 2020, 8, 3423. 32022089 10.1039/c9tb02474c

[9] S. C. Mukhopadhyay , IEEE Sens. J. 2015, 15, 1321.

[10] M. Amjadi , K. U. Kyung , I. Park , M. Sitti , Adv. Funct. Mater. 2016, 26, 1678.

[11] Y. Yang , W. Gao , Chem. Soc. Rev. 2019, 48, 1465. 29611861 10.1039/c7cs00730b

[12] J. Heikenfeld , A. Jajack , J. Rogers , P. Gutruf , L. Tian , T. Pan , R. Li , M. Khine , J. Kim , J. Wang , J. Kim , Lab Chip 2018, 18, 217. 29182185 10.1039/c7lc00914c PMC5771841

[13] B. Peng , F. Zhao , J. Ping , Y. Ying , Small 2020, 16, 2002681. 10.1002/smll.202002681 32893485

[14] W. A. D. M. Jayathilaka , K. Qi , Y. Qin , A. Chinnappan , W. Serrano‐García , C. Baskar , H. Wang , J. He , S. Cui , S. W. Thomas , S. Ramakrishna , Adv. Mater. 2019, 31, 1805921. 10.1002/adma.201805921 30589117

[15] S. Yao , P. Swetha , Y. Zhu , Adv. Healthcare Mater. 2018, 7, 1700889. 10.1002/adhm.201700889 29193793

[16] M. Singh , S. Meenu , S. Sayanti , B. Mayank , Y. Pragzna , D. Bommi , Int. J. Pharm. Biol. Arch. 2014, 5, 1.

[17] J. Heikenfeld , A. Jajack , B. Feldman , S. W. Granger , S. Gaitonde , G. Begtrup , B. A. Katchman , Nat. Biotechnol. 2019, 37, 407. 30804536 10.1038/s41587-019-0040-3

[18] A. C. Müller , F. P. Breitwieser , H. Fischer , C. Schuster , O. Brandt , J. Colinge , G. Superti‐Furga , G. Stingl , A. Elbe‐Bürger , K. L. Bennett , J. Proteome Res. 2012, 11, 3715. 22578099 10.1021/pr3002035

[19] J. Kool , L. Reubsaet , F. Wesseldijk , R. T. Maravilha , M. W. Pinkse , C. S. D'Santos , J. J. van Hilten , F. J. Zijlstra , A. J. R. Heck , Proteomics 2007, 7, 3638. 17890648 10.1002/pmic.200600938

[20] P. R. Miller , R. M. Taylor , B. Q. Tran , G. Boyd , T. Glaros , V. H. Chavez , R. Krishnakumar , A. Sinha , K. Poorey , K. P. Williams , S. S. Branda , J. T. Baca , R. Polsky , Commun. Biol. 2018, 1, 173. 30374463 10.1038/s42003-018-0170-z PMC6197253

[21] J. Madden , C. O'Mahony , M. Thompson , A. O'Riordan , P. Galvin , Sens. Bio‐Sens. Res. 2020, 29, 100348.

[22] S. A. N. Gowers , D. M. E. Freeman , T. M. Rawson , M. L. Rogers , R. C. Wilson , A. H. Holmes , A. E. Cass , D. O'Hare , ACS Sens. 2019, 4, 1072. 30950598 10.1021/acssensors.9b00288

[23] S. Sharma , A. El‐Laboudi , M. Reddy , N. Jugnee , S. Sivasubramaniyam , M. El Sharkawy , P. Georgiou , D. Johnston , N. Oliver , A. E. G. Cass , Anal. Methods 2018, 10, 2088.

[24] S. W. Ryter , A. M. K. Choi , J. Breath Res. 2013, 7, 017111. 23446063 10.1088/1752-7155/7/1/017111 PMC3651886

[25] S. A. Kharitonov , P. J. Barnes , Am. J. Respir. Crit. Care Med. 2001, 163, 1693. 11401895 10.1164/ajrccm.163.7.2009041

[26] Y. Qiao , Z. Gao , Y. Liu , Y. Liu , Y. Cheng , M. Yu , L. Zhao , Y. Duan , Biomed Res. Int. 2014, 2014, 869186. 24900994 10.1155/2014/869186 PMC4037575

[27] N. Alkhouri , T. Singh , E. Alsabbagh , J. Guirguis , T. Chami , I. Hanouneh , D. Grove , R. Lopez , R. Dweik , Clin. Transl. Gastroenterol. 2015, 6, 112. 10.1038/ctg.2015.40 PMC4816250 26378385

[28] W. Li , Y. Liu , Y. Liu , S. Cheng , Y. Duan , RSC Adv. 2017, 7, 17480.

[29] P. Martínez‐Lozano , J. F. De , La Mora , Anal. Chem. 2008, 80, 8210. 18821733 10.1021/ac801185e

[30] S. A. Kharitonov , M. Corradi , L. van Rensen , D. M. Geddes , M. E. Hodson , P. J. Barnes , P. Montuschi , G. Ciabattoni , Thorax 2000, 55, 205. 10679539 10.1136/thorax.55.3.205 PMC1745696

[31] S. W. Harshman , B. A. Geier , A. V Qualley , L. A. Drummond , L. E. Flory , M. Fan , R. L. Pitsch , C. C. Grigsby , J. B. Phillips , J. A. Martin , J. Breath Res. 2017, 11, 047111. 29018179 10.1088/1752-7163/aa927d

[32] Y. H. Ngo , M. Brothers , J. A. Martin , C. C. Grigsby , K. Fullerton , R. R. Naik , S. S. Kim , ACS Omega 2018, 3, 6230. 31458805 10.1021/acsomega.8b01039 PMC6644726

[33] F. Pabst , W. Miekisch , P. Fuchs , S. Kischkel , J. K. Schubert , J. Cardiothorac. Surg. 2007, 2, 1. 17877828 10.1186/1749-8090-2-37 PMC2100047

[34] M. Maniscalco , G. De Laurentiis , C. Pentella , M. Mormile , A. Sanduzzi , M. Sofia , G. De Laurentiis , C. Pentella , M. Mormile , A. Sanduzzi , Biomarkers 2006, 11, 233. 16760132 10.1080/13547500600692992

[35] A. Y. Kim , E. H. Jang , K. W. Choi , H. J. Jeon , S. Byun , J. Y. Sim , J. H. Choi , H. Y. Yu , PLoS One 2019, 14, e0213140. 30943195 10.1371/journal.pone.0213140 PMC6447153

[36] Z. Adams , E. A. McClure , K. M. Gray , C. K. Danielson , F. Treiber , K. J. Ruggiero , J. Psychiatr. Res. 2017, 85, 1. 27814455 10.1016/j.jpsychires.2016.10.019 PMC5191962

[37] W. Liu , Z. Lian , Q. Deng , Y. Liu , Build. Environ. 2011, 46, 478.

[38] S. Takada , S. Matsumoto , T. Matsushita , Build. Environ. 2013, 68, 123.

[39] M. Benedek , C. Kaernbach , J. Neurosci. Methods 2010, 190, 80. 20451556 10.1016/j.jneumeth.2010.04.028 PMC2892750

[40] W. Boucsein , D. C. Fowles , S. Grimnes , G. Ben‐Shakhar , W. T. Roth , M. E. Dawson , D. L. Filion , Psychophysiology 2012, 49, 1017. 22680988 10.1111/j.1469-8986.2012.01384.x

[41] S. D. Kreibig , F. H. Wilhelm , W. T. Roth , J. J. Gross , Psychophysiology 2007, 44, 787. 17598878 10.1111/j.1469-8986.2007.00550.x

[42] O. Grewe , R. Kopiez , E. Altenmüller , Music Percept. 2009, 27, 61.

[43] M. Benedek , C. Kaernbach , Biol. Psychol. 2011, 86, 320. 21276827 10.1016/j.biopsycho.2010.12.012 PMC3061318

[44] Y. Zhang , M. Haghdan , K. S. Xu , in Proc. – Int. Symp. Wearable Comput. ISWC, Association For Computing Machinery, New York 2017, pp. 54–57.

[45] A. Firooz , B. Sadr , S. Babakoohi , M. Sarraf‐Yazdy , F. Fanian , A. Kazerouni‐Timsar , M. Nassiri‐Kashani , M. M. Naghizadeh , Y. Dowlati , Sci. World J. 2012, 2012, 386936. 10.1100/2012/386936 PMC3317612 22536139

[46] D. Segger , A. Matthies , T. Saldeen , J. Dermatolog. Treat. 2008, 19, 279. 19160533 10.1080/09546630801958238

[47] S. Yoon , J. K. Sim , N. Park , Y. H. Cho , Sci. Rep. 2018, 8, 23468.

[48] F. McLafferty , C. Hendry , F. Alistair , Nurs. Stand. 2012, 27, 35. 10.7748/ns2012.10.27.7.35.c9358 23248884

[49] J. Kim , D. G. Seo , Y. H. Cho , Appl. Phys. Lett. 2014, 104, 253502.

[50] I. Doh , H. K. Lim , B. Ahn , Metrologia 2015, 52, 291.

[51] Y. Ma , J. Choi , A. Hourlier‐Fargette , Y. Xue , H. U. Chung , J. Y. Lee , X. Wang , Z. Xie , D. Kang , H. Wang , S. Han , S. K. Kang , Y. Kang , X. Yu , M. J. Slepian , M. S. Raj , J. B. Model , X. Feng , R. Ghaffari , J. A. Rogers , Y. Huang , Proc. Natl. Acad. Sci. USA 2018, 115, 11144. 30322935 10.1073/pnas.1814392115 PMC6217416

[52] J. Allen , Physiol. Meas. 2007, 28, R1. 17322588 10.1088/0967-3334/28/3/R01

[53] E. Gil , M. Orini , R. Bailón , J. M. Vergara , L. Mainardi , P. Laguna , Physiol. Meas. 2010, 31, 1271. 20702919 10.1088/0967-3334/31/9/015

[54] K. Wilke , A. Martin , L. Terstegen , S. S. Biel , Int. J. Cosmet. Sci. 2007, 29, 169. 18489347 10.1111/j.1467-2494.2007.00387.x

[55] S. N. Cheuvront , S. E. Bearden , R. W. Kenefick , B. R. Ely , D. W. DeGroot , M. N. Sawka , S. J. Montain , J. Appl. Physiol. 2009, 107, 69. 19423839 10.1152/japplphysiol.00250.2009

[56] S. Chen , M. H. Shamsi , J. Micromech. Microeng. 2017, 27, 083001.

[57] D. Ji , Z. Shi , Z. Liu , S. S. Low , J. Zhu , T. Zhang , Z. Chen , X. Yu , Y. Lu , D. Lu , Q. Liu , Smart Mater. Med. 2020, 1, 1.

[58] A. Ainla , M. P. S. Mousavi , M. N. Tsaloglou , J. Redston , J. G. Bell , M. T. Fernández‐Abedul , G. M. Whitesides , Anal. Chem. 2018, 90, 6240. 29658268 10.1021/acs.analchem.8b00850 PMC5997382

[59] L. Sun , C. Sun , X. Sun , Electrochim. Acta 2016, 220, 690.

[60] S. Lifson , C. E. Felder , A. Shanzer , J. Am. Chem. Soc. 1983, 105, 3866.

[61] P. Bühlmann , L. D. Chen , Supramolecular Chemistry, John Wiley & Sons, Ltd, Chichester, UK 2012.

[62] P. Bühlmann , L. D. Chen Ion‐Selective Electrodes With Ionophore‐Doped Sensing Membranes, Hoboken, New Jersey 2012.

[63] S. Wang , Y. Wu , Y. Gu , T. Li , H. Luo , L.‐H. Li , Y. Bai , L. Li , L. Liu , Y. Cao , H. Ding , T. Zhang , Anal. Chem. 2017, 89, 10224. 28862001 10.1021/acs.analchem.7b01560

[64] D. P. Rose , M. E. Ratterman , D. K. Griffin , L. Hou , N. Kelley‐Loughnane , R. R. Naik , J. A. Hagen , I. Papautsky , J. C. Heikenfeld , IEEE Trans. Biomed. Eng. 2015, 62, 1457. 25398174 10.1109/TBME.2014.2369991

[65] H. M. I. Salzitsa Anastasova , Blair Crewther , Pawel Bembnowicz , Vincenzo Curto , G.‐Z. Y. Bruno Rosa , Biosens. Bioelectron. 2017, 93, 139. 27743863 10.1016/j.bios.2016.09.038

[66] W. Gao , S. Emaminejad , H. Y. Y. Nyein , S. Challa , K. Chen , A. Peck , H. M. Fahad , H. Ota , H. Shiraki , D. Kiriya , D. H. Lien , G. A. Brooks , R. W. Davis , A. Javey , Nature 2016, 529, 509. 26819044 10.1038/nature16521 PMC4996079

[67] H. Y. Y. Nyein , W. Gao , Z. Shahpar , S. Emaminejad , S. Challa , K. Chen , H. M. Fahad , L.‐C. Tai , H. Ota , R. W. Davis , A. Javey , ACS Nano 2016, 10, 7216. 27380446 10.1021/acsnano.6b04005

[68] H. Lee , C. Song , Y. S. Hong , M. S. Kim , H. R. Cho , T. Kang , K. Shin , S. H. Choi , T. Hyeon , D. Kim , Sci. Adv. 2017, 3, e1601314. 28345030 10.1126/sciadv.1601314 PMC5342654

[69] S. Anastasova , B. Crewther , P. Bembnowicz , V. Curto , H. M. Ip , B. Rosa , G. Z. Yang , Biosens. Bioelectron. 2017, 94, 730. 27743863 10.1016/j.bios.2016.09.038

[70] M. Campàs , B. Prieto‐Simón , J. L. Marty , Semin. Cell Dev. Biol. 2009, 20, 3. 19429486 10.1016/j.semcdb.2009.01.009

[71] S. Ferri , K. Kojima , K. Sode , J. Diabetes Sci. Technol. 2011, 5, 1068. 22027299 10.1177/193229681100500507 PMC3208862

[72] H. Kumar , R. Neelam , Nanobiosens. Dis. Diagn. 2016, 5, 29.

[73] C. Zhu , G. Yang , H. Li , D. Du , Y. Lin , C. Zhu , G. Yang , H. Li , D. Du , Y. Lin , Anal. Chem. 2015, 87, 230. 25354297 10.1021/ac5039863 PMC4287168

[74] E. Ö. Bolat , G. A. Tığ , Ş. Pekyardımcı , J. Electroanal. Chem. 2017, 785, 241.

[75] W. Gao , S. Emaminejad , H. Y. Y. Nyein , S. Challa , K. Chen , A. Peck , H. M. Fahad , H. Ota , H. Shiraki , D. Kiriya , D. H. Lien , G. A. Brooks , R. W. Davis , A. Javey , Nature 2016, 529, 509. 26819044 10.1038/nature16521 PMC4996079

[76] J. Kim , I. Jeerapan , S. Imani , T. N. Cho , A. Bandodkar , S. Cinti , P. P. Mercier , J. Wang , ACS Sens. 2016, 1, 1011.

[77] A. Brueck , T. Iftekhar , A. Stannard , K. Yelamarthi , T. Kaya , Sensors 2018, 18, 533. 10.3390/s18020533 PMC5855985 29439398

[78] T. M. Squires , R. J. Messinger , S. R. Manalis , Nat. Biotechnol. 2008, 26, 417. 18392027 10.1038/nbt1388

[79] T. M. Squires , S. R. Quake , Rev. Mod. Phys. 2005, 77, 977.

[80] I. Bazin , S. A. Tria , A. Hayat , J. L. Marty , Biosens. Bioelectron. 2017, 87, 285. 27568847 10.1016/j.bios.2016.06.083

[81] X. Xiao , Z. Kuang , J. M. Slocik , S. Tadepalli , M. Brothers , S. Kim , P. A. Mirau , C. Butkus , B. L. Farmer , S. Singamaneni , C. K. Hall , R. R. Naik , ACS Sens. 2018, 3, 1024. 29741092 10.1021/acssensors.8b00159

[82] L. Gold , D. Ayers , J. Bertino , C. Bock , A. Bock , E. N. Brody , J. Carter , A. B. Dalby , B. E. Eaton , T. Fitzwater , D. Flather , A. Forbes , T. Foreman , C. Fowler , B. Gawande , M. Goss , M. Gunn , S. Gupta , D. Halladay , J. Heil , J. Heilig , B. Hicke , G. Husar , N. Janjic , T. Jarvis , S. Jennings , E. Katilius , T. R. Keeney , N. Kim , T. H. Koch , et al., PLoS One 2010, 5, 15004. 10.1371/journal.pone.0015004 PMC3000457 21165148

[83] M. Kimoto , R. S. Cox , I. Hirao , Expert Rev. Mol. Diagn. 2011, 11, 321. 21463241 10.1586/erm.11.5

[84] M. F. Kubik , C. Bell , T. Fitzwater , S. R. Watson , D. M. Tasset , J. Immunol. 1997, 159, 259. 9200462

[85] M. J. Kujau , S. Wölfl , Nucleic Acids Res. 1998, 26, 1851. 9512563 10.1093/nar/26.7.1851 PMC147467

[86] N. Misawa , T. Osaki , S. Takeuchi , J. R. Soc., Interface 2018, 15, 20170952. 29669891 10.1098/rsif.2017.0952 PMC5938585

[87] E. P. Carpenter , K. Beis , A. D. Cameron , S. Iwata , Curr. Opin. Struct. Biol. 2008, 18, 581. 18674618 10.1016/j.sbi.2008.07.001 PMC2580798

[88] O. Y. Dmitriev , S. Lutsenko , S. Muyldermans , J. Biol. Chem. 2016, 291, 3767. 26677230 10.1074/jbc.R115.679811 PMC4759159

[89] R. M. Torrente‐Rodríguez , J. Tu , Y. Yang , J. Min , M. Wang , Y. Song , Y. Yu , C. Xu , C. Ye , W. W. IsHak , W. Gao , Matter 2020, 2, 921. 32266329 10.1016/j.matt.2020.01.021 PMC7138219

[90] N. Phares , R. J. White , K. W. Plaxco , Anal. Chem. 2009, 81, 1095. 19133790 10.1021/ac8021983 PMC3956047

[91] S. N. Kim , Z. Kuang , J. M. Slocik , S. E. Jones , Y. Cui , B. L. Farmer , M. C. McAlpine , R. R. Naik , J. Am. Chem. Soc. 2011, 133, 14480. 21861527 10.1021/ja2042832

[92] C. Muratore , A. T. Juhl , A. J. Stroud , D. Wenbi Lai , A. M. Jawaid , K. M. Burzynski , J. M. Dagher , G. M. Leuty , C. Harsch , S. S. Kim , Y. H. Ngo , N. R. Glavin , R. J. Berry , M. F. Durstock , P. A. Derosa , A. K. Roy , E. M. Heckman , R. R. Naik , Appl. Phys. Lett. 2018, 112, 233704.

[93] R. B. Pandey , Z. Kuang , B. L. Farmer , S. S. Kim , R. R. Naik , Soft Matter 2012, 8, 9101.

[94] S. Balamurugan , A. Obubuafo , S. A. Soper , D. A. Spivak , Anal. Bioanal. Chem. 2008, 390, 1009. 17891385 10.1007/s00216-007-1587-2

[95] A. E. Strong , B. D. Moore , J. Mater. Chem. 1999, 9, 1097.

[96] N. R. Mohamad , N. H. C. Marzuki , N. A. Buang , F. Huyop , R. A. Wahab , Biotechnol. Biotechnol. Equip. 2015, 29, 205. 26019635 10.1080/13102818.2015.1008192 PMC4434042

[97] W. Putzbach , N. J. Ronkainen , Sensors 2013, 13, 4811. 23580051 10.3390/s130404811 PMC3673113

[98] Z. Kuang , S. N. Kim , W. J. Crookes‐goodson , B. L. Farmer , R. R. Naik , ACS Nano 2010, 4, 452. 20038158 10.1021/nn901365g

[99] A. De Andrade Fernandes , P. R. Dos Santos Amorim , C. J. Brito , A. G. De Moura , D. G. Moreira , C. M. A. Costa , M. Sillero‐Quintana , J. C. B. Marins , Physiol. Meas. 2014, 35, 189. 24398429 10.1088/0967-3334/35/2/189

[100] J. K. Sim , S. Youn , Y. H. Cho , J. Micromech. Microeng. 2012, 22, 125014.

[101] J. K. Sim , Y. H. Cho , J. Nanosci. Nanotechnol. 2016, 16, 4422. 27483767 10.1166/jnn.2016.11003

[102] J. Ogorevc , G. Geršak , D. Novak , J. Drnovšek , Meas.: J. Int. Meas. Confed. 2013, 46, 2993.

[103] M. A. Kim , E. J. Kim , H. K. Lee , Ski. Res. Technol. 2018, 24, 466. 10.1111/srt.12455 29405450

[104] M. R. Burcher , J. A. Noble , L. Han , M. Gooding , IEEE Trans. Ultrason. Ferroelectr. Freq. Control 2005, 52, 1330. 16245602 10.1109/tuffc.2005.1509791

[105] W. Yu , Y. Li , Y. P. Zheng , N. Y. Lim , M. H. Lu , J. Fan , Meas. Sci. Technol. 2006, 17, 1785.

[106] T. Vandenberk , J. Stans , C. Mortelmans , R. Van Haelst , G. Van Schelvergem , C. Pelckmans , C. J. Smeets , D. Lanssens , H. De Cannière , V. Storms , I. M. Thijs , B. Vaes , P. M. Vandervoort , JMIR mHealth uHealth 2017, 5, 129. 10.2196/mhealth.7254 PMC5591405 28842392

[107] E. Gassmann , Introduction to Pressure Measurement, New York, NY 2014.

[108] P. Palatini , Sports Med. 1988, 5, 353. 3041529 10.2165/00007256-198805060-00002

[109] J. Nuutinen , E. Alanen , P. Autio , M.‐R. Lahtinen , I. Harvima , T. Lahtinen , Ski. Res. Technol. 2003, 9, 85. 10.1034/j.1600-0846.2003.00025.x 12709124

[110] M. Mündlein , B. Valentin , R. Chabicovsky , J. Nicolics , J. Weremczuk , G. Tarapata , R. Jachowicz , Sens. Actuators, A 2008, 142, 67.

[111] R. E. Imhof , M. E. P. De Jesus , P. Xiao , L. I. Ciortea , E. P. Berg , Int. J. Cosmet. Sci. 2009, 31, 97. 19175433 10.1111/j.1468-2494.2008.00476.x

[112] J. Choi , R. Ghaffari , L. B. Baker , J. A. Rogers , Sci. Adv. 2018, 4, eaar3921. 29487915 10.1126/sciadv.aar3921 PMC5817925

[113] J. Francis , I. Stamper , J. Heikenfeld , E. F. Gomez , Lab Chip 2018, 19, 178. 30525141 10.1039/c8lc00968f

[114] R. Cataldo , M. Leuzzi , E. Alfinito , Chemosensors 2018, 6, 20.

[115] A. E. Islam , R. Martineau , S. S. Kim , B. Maruyama , L. F. Drummy , Analytical Methods, Royal Society of Chemistry, London 2013, pp. 1098–1115.

[116] Y. Xiao , A. A. Lubin , A. J. Heeger , K. W. Plaxco , Angew. Chem., Int. Ed. 2005, 44, 5456. 10.1002/anie.200500989 16044476

[117] N. Aliakbarinodehi , P. Jolly , N. Bhalla , A. Miodek , G. De Micheli , P. Estrela , S. Carrara , Sci. Rep. 2017, 7, 44409. 28294122 10.1038/srep44409 PMC5353720

[118] H. Li , J. Somerson , F. Xia , K. W. Plaxco , Anal. Chem. 2018, 90, 10641. 30141321 10.1021/acs.analchem.8b01993 PMC6555152

[119] H. Li , P. Dauphin‐Ducharme , G. Ortega , K. W. Plaxco , J. Am. Chem. Soc. 2017, 139, 11207. 28712286 10.1021/jacs.7b05412 PMC6519131

[120] F. Ricci , K. W. Plaxco , Microchimica. Acta. 2008, 163, 149.

[121] R. J. White , A. A. Rowe , K. W. Plaxco , Analyst 2010, 135, 589. 20174715 10.1039/b921253a PMC2861036

[122] N. Arroyo‐Currás , J. Somerson , P. A. Vieira , K. L. Ploense , T. E. Kippin , K. W. Plaxco , Proc. Natl. Acad. Sci. USA 2017, 114, 645. 28069939 10.1073/pnas.1613458114 PMC5278471

[123] H. Li , P. Dauphin‐Ducharme , N. Arroyo‐Currás , C. H. Tran , P. A. Vieira , S. Li , C. Shin , J. Somerson , T. E. Kippin , K. W. Plaxco , Angew. Chem., Int. Ed. 2017, 56, 7492. 10.1002/anie.201700748 PMC5660315 28371090

[124] J. Luo , S. Jiang , H. Zhang , J. Jiang , X. Liu , Anal. Chim. Acta 2012, 709, 47. 22122930 10.1016/j.aca.2011.10.025

[125] T. Hianik , V. Ostatná , M. Sonlajtnerova , I. Grman , Bioelectrochemistry 2007, 70, 127. 16725379 10.1016/j.bioelechem.2006.03.012

[126] Ni̇. Demİrkiran , E. Ekİncİ , M. Asİltürk , 2012, 4, 3.

[127] G. Seo , G. Lee , M. J. Kim , S.‐H. Baek , M. Choi , K. B. Ku , C.‐S. Lee , S. Jun , D. Park , H. G. Kim , S.‐J. Kim , J.‐O. Lee , B. T. Kim , E. C. Park , S. Il Kim , ACS Nano 2020, 14, 5135. 32293168 10.1021/acsnano.0c02823

[128] Z. Yi , J. Sayago , Different Types Field‐Effect Transistors: Theory and Applications, InTechOpen, London, UK 2017, pp. 165–181.

[129] P. Bergveld , Sens. Actuators 1981, 1, 17.

[130] E. Stern , J. F. Klemic , D. A. Routenberg , P. N. Wyrembak , D. B. Turner‐Evans , A. D. Hamilton , D. A. LaVan , T. M. Fahmy , M. A. Reed , Nature 2007, 445, 519. 17268465 10.1038/nature05498

[131] P. R. Nair , M. A. Alam , Appl. Phys. Lett. 2006, 88, 233120.

[132] G. S. Kulkarni , Z. Zhong , Nano Lett. 2012, 12, 719. 22214376 10.1021/nl203666a

[133] A. E. Islam , R. Martineau , S. S. Kim , B. Maruyama , L. F. Drummy , SISC 2019 50th IEEE Semiconductor Interface Specialists Conf., 2019, pp. 177–178.

[134] M. A. Brown , Z. Abbas , A. Kleibert , R. G. Green , A. Goel , S. May , T. M. Squires , Phys. Rev. X 2016, 6, 011007.

[135] A. E. Islam , A. E. Islam , R. Martineau , R. Martineau , C. M. Crasto , C. M. Crasto , H. Kim , R. S. Rao , R. S. Rao , B. Maruyama , S. S. Kim , L. F. Drummy , ACS Appl. Nano Mater. 2020, 3, 5088.

[136] J. Ping , J. Xi , J. G. Saven , R. Liu , A. T. C. Johnson , Biosens. Bioelectron. 2017, 89, 689. 26626969 10.1016/j.bios.2015.11.052 PMC4873466

[137] E. Piccinini , S. Alberti , G. S. Longo , T. Berninger , J. Breu , J. Dostalek , O. Azzaroni , W. Knoll , J. Phys. Chem. C 2018, 122, 10181.

[138] N. Gao , T. Gao , X. Yang , X. Dai , W. Zhou , A. Zhang , C. M. Lieber , Proc. Natl. Acad. Sci. USA 2016, 113, 14633. 27930344 10.1073/pnas.1625010114 PMC5187689

[139] D. Sarkar , W. Liu , X. Xie , A. C. Anselmo , S. Mitragotri , K. Banerjee , ACS Nano 2014, 8, 3992. 24588742 10.1021/nn5009148

[140] Y. Montagut , J. V. García , Y. Jiménez , C. March , Á. Montoya , A. Arnau , Sensors 2011, 11, 4702. 22163871 10.3390/s110504702 PMC3231406

[141] A. Sabdo Yuwono , P. Schulze Lammers , Odor Pollution in the Environment and the Detection Instrumentation, Beijing, China 2004.

[142] J. L. Casteleiro‐Roca , J. L. Calvo‐Rolle , M. C. Meizoso‐Lopez , A. Piñón‐Pazos , B. A. Rodríguez‐Gómez , Sens. Actuators, A 2014, 207, 1.

[143] S. Heydari , G. H. Haghayegh , J. Sens. Technol. 2014, 04, 81.

[144] T. Ito , N. Aoki , S. Kaneko , K. Suzuki , Anal. Methods 2014, 6, 7469. 10.1039/c4ay01387e 40883939

[145] G. Bayramoglu , C. Ozalp , M. Oztekin , U. Guler , B. Salih , M. Y. Arica , Talanta 2019, 191, 59. 30262099 10.1016/j.talanta.2018.08.048

[146] Z. M. Dong , L. Cheng , P. Zhang , G. C. Zhao , Analyst 2020, 145, 3329. 32207499 10.1039/d0an00308e

[147] J. Fritz , Analyst 2008, 133, 855. 18575634 10.1039/b718174d

[148] B. N. Johnson , R. Mutharasan , Biosens. Bioelectron. 2012, 32, 1. 22119230 10.1016/j.bios.2011.10.054

[149] M. A. Cooper , Nat. Rev. Drug Discovery 2002, 1, 515. 12120258 10.1038/nrd838

[150] M. Maute , S. Raible , F. E. Prins , D. P. Kern , U. Weimar , W. Göpel , Microelectron. Eng. 1999, 46, 439.

[151] A. K. Basu , A. Basu , S. Bhattacharya , Enzyme Microb. Technol. 2020, 139, 109558. 32732024 10.1016/j.enzmictec.2020.109558

[152] S. T. Koev , M. A. Powers , H. Yi , L. Q. Wu , W. E. Bentley , G. W. Rubloff , G. F. Payne , R. Ghodssi , Lab Chip 2007, 7, 103. 17180212 10.1039/b609149k

[153] Y. Arntz , J. D. Seelig , H. P. Lang , J. Zhang , P. Hunziker , J. P. Ramseyer , E. Meyer , M. Hegner , C. Gerber , Nanotechnology 2003, 14, 86.

[154] S. Stassi , E. Fantino , R. Calmo , A. Chiappone , M. Gillono , D. Scaiola , C. F. Pirri , C. Ricciardi , A. Chiadò , I. Roppolo , ACS Appl. Mater. Interfaces 2017, 9, 19193. 28530385 10.1021/acsami.7b04030

[155] J. R. Mejía‐Salazar , O. N. Oliveira , Chem. Rev. 2018, 118, 10617. 30247025 10.1021/acs.chemrev.8b00359

[156] J. F. Masson , Analyst 2020, 145, 3776. 32374303 10.1039/d0an00316f

[157] Z. Wei , Z.‐K. Zhou , Q. Li , J. Xue , A. Di Falco , Z. Yang , J. Zhou , X. Wang , Small 2017, 13, 1700109. 10.1002/smll.201700109 28544454

[158] K. Park , M. S. Hsiao , Y. J. Yi , S. Izor , H. Koerner , A. Jawaid , R. A. Vaia , ACS Appl. Mater. Interfaces 2017, 9, 26363. 28714667 10.1021/acsami.7b08003

[159] A. L. Schmucker , S. Tadepalli , K. K. Liu , C. J. Sullivan , S. Singamaneni , R. R. Naik , RSC Adv. 2016, 6, 4136.

[160] A. Abbas , A. Brimer , J. M. Slocik , L. Tian , R. R. Naik , S. Singamaneni , Anal. Chem. 2013, 85, 3977. 23425068 10.1021/ac303567g

[161] M. Loyez , J. C. Larrieu , S. Chevineau , M. Remmelink , D. Leduc , B. Bondue , P. Lambert , J. Devière , R. Wattiez , C. Caucheteur , Biosens. Bioelectron. 2019, 131, 104. 30826644 10.1016/j.bios.2019.01.062

[162] D. Li , J. Wu , P. Wu , Y. Lin , Y. Sun , R. Zhu , J. Yang , K. Xu , Sens. Actuators, B 2015, 213, 295.

[163] H. SadAbadi , S. Badilescu , M. Packirisamy , R. Wüthrich , Biosens. Bioelectron. 2013, 44, 77. 23395726 10.1016/j.bios.2013.01.016

[164] A. Koh , D. Kang , Y. Xue , S. Lee , R. M. Pielak , J. Kim , T. Hwang , S. Min , A. Banks , P. Bastien , M. C. Manco , L. Wang , K. R. Ammann , K. I. Jang , P. Won , S. Han , R. Ghaffari , U. Paik , M. J. Slepian , G. Balooch , Y. Huang , J. A. Rogers , Sci. Transl. Med. 2016, 8, 366ra165. 10.1126/scitranslmed.aaf2593 PMC5429097 27881826

[165] H. Araki , J. Kim , S. Zhang , A. Banks , K. E. Crawford , X. Sheng , P. Gutruf , Y. Shi , R. M. Pielak , J. A. Rogers , Adv. Funct. Mater. 2017, 27, 1604465.

[166] L. Yu , N. Li , Chemosensors 2019, 7, 53.

[167] V. X. T. Zhao , T. I. Wong , X. T. Zheng , Y. N. Tan , X. Zhou , Mater. Sci. Energy Technol. 2020, 3, 237. 33604529 10.1016/j.mset.2019.10.002 PMC7148662

[168] J. Choi , Y. Xue , W. Xia , T. R. Ray , J. T. Reeder , A. J. Bandodkar , D. Kang , S. Xu , Y. Huang , J. A. Rogers , Lab Chip 2017, 17, 2572. 28664954 10.1039/c7lc00525c PMC5561737

[169] S. B. Kim , Y. Zhang , S. M. Won , A. J. Bandodkar , Y. Sekine , Y. Xue , J. Koo , S. W. Harshman , J. A. Martin , J. M. Park , T. R. Ray , K. E. Crawford , K. T. Lee , J. Choi , R. L. Pitsch , C. C. Grigsby , A. J. Strang , Y. Y. Chen , S. Xu , J. Kim , A. Koh , J. S. Ha , Y. Huang , S. W. Kim , J. A. Rogers , Small 2018, 14, 1703334.

[170] D. H. Kim , N. Lu , Y. Huang , J. A. Rogers , MRS Bull. 2012, 37, 226.

[171] Y. Zhang , R. C. Webb , H. Luo , Y. Xue , J. Kurniawan , N. H. Cho , S. Krishnan , Y. Li , Y. Huang , J. A. Rogers , Adv. Healthcare Mater. 2016, 5, 119. 10.1002/adhm.201670002 26749418

[172] R. C. Webb , R. M. Pielak , P. Bastien , J. Ayers , J. Niittynen , J. Kurniawan , M. Manco , A. Lin , N. H. Cho , V. Malyrchuk , G. Balooch , J. A. Rogers , PLoS One 2015, 10, 0118131. 10.1371/journal.pone.0118131 PMC4319855 25658947

[173] S. R. Madhvapathy , Y. Ma , M. Patel , S. Krishnan , C. Wei , Y. Li , S. Xu , X. Feng , Y. Huang , J. A. Rogers , Adv. Funct. Mater. 2018, 28, 1870242.

[174] X. Huang , W. H. Yeo , Y. Liu , J. A. Rogers , Biointerphases 2012, 7, 52. 22915327 10.1007/s13758-012-0052-8

[175] S. Krishnan , Y. Shi , R. C. Webb , Y. Ma , P. Bastien , K. E. Crawford , A. Wang , X. Feng , M. Manco , J. Kurniawan , E. Tir , Y. Huang , G. Balooch , R. M. Pielak , J. A. Rogers , Microsyst. Nanoeng. 2017, 3, 17014. 31057861 10.1038/micronano.2017.14 PMC6444991

[176] X. Huang , Y. Liu , H. Cheng , W. J. Shin , J. A. Fan , Z. Liu , C. J. Lu , G. W. Kong , K. Chen , D. Patnaik , S. H. Lee , S. Hage‐Ali , Y. Huang , J. A. Rogers , Adv. Funct. Mater. 2014, 24, 3846.

[177] J. Choi , A. J. Bandodkar , J. T. Reeder , T. R. Ray , A. Turnquist , S. B. Kim , N. Nyberg , A. Hourlier‐Fargette , J. B. Model , A. J. Aranyosi , S. Xu , R. Ghaffari , J. A. Rogers , ACS Sens. 2019, 4, 379. 30707572 10.1021/acssensors.8b01218

[178] X. Huang , Y. Liu , K. Chen , W. J. Shin , C. J. Lu , G. W. Kong , D. Patnaik , S. H. Lee , J. F. Cortes , J. A. Rogers , Small 2014, 10, 3083. 24706477 10.1002/smll.201400483

[179] S. B. Kim , K. H. Lee , M. S. Raj , B. Lee , J. T. Reeder , J. Koo , A. Hourlier‐Fargette , A. J. Bandodkar , S. M. Won , Y. Sekine , J. Choi , Y. Zhang , J. Yoon , B. H. Kim , Y. Yun , S. Lee , J. Shin , J. Kim , R. Ghaffari , J. A. Rogers , Small 2018, 14, 1802876. 10.1002/smll.201802876 30300469

[180] J. Kim , G. A. Salvatore , H. Araki , A. M. Chiarelli , Z. Xie , A. Banks , X. Sheng , Y. Liu , J. W. Lee , K. I. Jang , S. Y. Heo , K. Cho , H. Luo , B. Zimmerman , J. Kim , L. Yan , X. Feng , S. Xu , M. Fabiani , G. Gratton , Y. Huang , U. Paik , J. A. Rogers , Sci. Adv. 2016, 2, e1600418. 27493994 10.1126/sciadv.1600418 PMC4972468

[181] R. C. Webb , Y. Ma , S. Krishnan , Y. Li , S. Yoon , X. Guo , X. Feng , Y. Shi , M. Seidel , N. H. Cho , J. Kurniawan , J. Ahad , N. Sheth , J. Kim , J. G. TaylorVI , T. Darlington , K. Chang , W. Huang , J. Ayers , A. Gruebele , R. M. Pielak , M. J. Slepian , Y. Huang , A. M. Gorbach , J. A. Rogers , Sci. Adv. 2015, 1, e1500701. 26601309 10.1126/sciadv.1500701 PMC4646823

[182] S. Han , J. Kim , S. M. Won , Y. Ma , D. Kang , Z. Xie , K. T. Lee , H. U. Chung , A. Banks , S. Min , S. Y. Heo , C. R. Davies , J. W. Lee , C. H. Lee , B. H. Kim , K. Li , Y. Zhou , C. Wei , X. Feng , Y. Huang , J. A. Rogers , Sci. Transl. Med. 2018, 10, eaan4950. 29618561 10.1126/scitranslmed.aan4950 PMC5996377

[183] J. Zhao , Y. Lin , J. Wu , H. Y. Y. Nyein , M. Bariya , L. C. Tai , M. Chao , W. Ji , G. Zhang , Z. Fan , A. Javey , ACS Sens. 2019, 4, 1925. 31271034 10.1021/acssensors.9b00891

[184] Y. Lin , M. Bariya , H. Y. Y. Nyein , L. Kivimäki , S. Uusitalo , E. Jansson , W. Ji , Z. Yuan , T. Happonen , C. Liedert , J. Hiltunen , Z. Fan , A. Javey , Adv. Funct. Mater. 2019, 29, 1.

[185] H. Y. Y. Nyein , L. C. Tai , Q. P. Ngo , M. Chao , G. B. Zhang , W. Gao , M. Bariya , J. Bullock , H. Kim , H. M. Fahad , A. Javey , ACS Sens. 2018, 3, 944. 29741360 10.1021/acssensors.7b00961

[186] H. Y. Y. Nyein , M. Bariya , L. Kivimäki , S. Uusitalo , T. S. Liaw , E. Jansson , C. H. Ahn , J. A. Hangasky , J. Zhao , Y. Lin , T. Happonen , M. Chao , C. Liedert , Y. Zhao , L. C. Tai , J. Hiltunen , A. Javey , Sci. Adv. 2019, 5, eaaw9906. 31453333 10.1126/sciadv.aaw9906 PMC6697435

[187] Y. Gao , H. Ota , E. W. Schaler , K. Chen , A. Zhao , W. Gao , H. M. Fahad , Y. Leng , A. Zheng , F. Xiong , C. Zhang , L. C. Tai , P. Zhao , R. S. Fearing , A. Javey , Adv. Mater. 2017, 29, 1701985. 10.1002/adma.201701985 28833673

[188] J. R. Windmiller , A. J. Bandodkar , G. Valdés‐Ramirez , S. Parkhomovsky , A. G. Martinez , J. Wang , Chem. Commun. 2012, 48, 6794. 10.1039/c2cc32839a PMC3387561 22669136

[189] A. J. Bandodkar , D. Molinnus , O. Mirza , T. Guinovart , J. R. Windmiller , G. Valdés‐Ramírez , F. J. Andrade , M. J. Schöning , J. Wang , Biosens. Bioelectron. 2014, 54, 603. 24333582 10.1016/j.bios.2013.11.039

[190] A. J. Bandodkar , W. Jia , C. Yardimci , X. Wang , J. Ramirez , J. Wang , Anal. Chem. 2015, 87, 394. 25496376 10.1021/ac504300n

[191] A. Abellán‐Llobregat , I. Jeerapan , A. Bandodkar , L. Vidal , A. Canals , J. Wang , E. Morallón , Biosens. Bioelectron. 2017, 91, 885. 28167366 10.1016/j.bios.2017.01.058 PMC5328638

[192] A. J. Bandodkar , S. Imani , R. Nuñez‐Flores , R. Kumar , C. Wang , A. M. V. Mohan , J. Wang , P. P. Mercier , Biosens. Bioelectron. 2018, 101, 181. 29073519 10.1016/j.bios.2017.10.019 PMC5841915

[193] J. Kim , A. S. Campbell , J. Wang , Talanta 2018, 177, 163. 29108571 10.1016/j.talanta.2017.08.077

[194] C. A. Silva , J. lv , L. Yin , I. Jeerapan , G. Innocenzi , F. Soto , Y. G. Ha , J. Wang , Adv. Funct. Mater. 2020, 30, 2002041.

[195] W. Jia , A. J. Bandodkar , G. Valdés‐Ramírez , J. R. Windmiller , Z. Yang , J. Ramírez , G. Chan , J. Wang , Anal. Chem. 2013, 85, 6553. 23815621 10.1021/ac401573r

[196] J. R. Sempionatto , A. A. Khorshed , A. Ahmed , A. N. De Loyola e Silva , A. Barfidokht , L. Yin , K. Y. Goud , M. A. Mohamed , E. Bailey , J. May , C. Aebischer , C. Chatelle , J. Wang , ACS Sens. 2020, 5, 1804. 32366089 10.1021/acssensors.0c00604

[197] M. Bariya , Z. Shahpar , H. Park , J. Sun , Y. Jung , W. Gao , H. Y. Y. Nyein , T. S. Liaw , L. C. Tai , Q. P. Ngo , M. Chao , Y. Zhao , M. Hettick , G. Cho , A. Javey , ACS Nano 2018, 12, 6978. 29924589 10.1021/acsnano.8b02505

[198] G. Iorgulescu , J. Med. Life 2009, 2, 303. 20112475 PMC5052503

[199] P. Simmers , S. K. Li , G. Kasting , J. Heikenfeld , J. Dermatol. Sci. 2017, 89, 40. 29128285 10.1016/j.jdermsci.2017.10.013

[200] J. H. Burn , J. Physiol. 1925, 60, 365. 16993761 10.1113/jphysiol.1925.sp002255 PMC1514754

[201] S. L. Davis , J. Appl. Physiol. 2005, 98, 1740. 15640392 10.1152/japplphysiol.00860.2004

[202] D. Vairo , L. Bruzzese , M. Marlinge , L. Fuster , N. Adjriou , N. Kipson , P. Brunet , J. Cautela , Y. Jammes , G. Mottola , S. Burtey , J. Ruf , R. Guieu , E. Fenouillet , Sci. Rep. 2017, 7, 11801. 28924220 10.1038/s41598-017-12211-y PMC5603548

[203] Y. Yang , W. Gao , Chem. Soc. Rev. 2018, 48, 1465. 10.1039/c7cs00730b 29611861

[204] J. A. Yagiela , Pharmacology and Therapeutics for Dentistry, Elsevier, Amsterdam, Netherlands 2011.

[205] A. Jajack , M. Brothers , G. Kasting , J. Heikenfeld , PLoS One 2018, 13, 0200009. 10.1371/journal.pone.0200009 PMC6047769 30011292

[206] M. Tierney , Biosens. Bioelectron. 2001, 16, 621. 11679237 10.1016/s0956-5663(01)00189-0

[207] M. Leonard , E. Creed , D. Brayden , A. W. Baird , Pharm. Res. 2000, 17, 476. 10870994 10.1023/a:1007541423500

[208] K. Natsuga , Cold Spring Harbor Perspect. Med. 2014, 4, 018218. 10.1101/cshperspect.a018218 PMC3968788 24692192

[209] P. P. Samant , M. R. Prausnitz , Proc. Natl. Acad. Sci. USA 2018, 115, 4583. 29666252 10.1073/pnas.1716772115 PMC5939066

[210] H. Teymourian , C. Moonla , F. Tehrani , E. Vargas , R. Aghavali , A. Barfidokht , T. Tangkuaram , P. P. Mercier , E. Dassau , J. Wang , Anal. Chem. 2020, 92, 2291. 31874029 10.1021/acs.analchem.9b05109

[211] P. R. Miller , X. Xiao , I. Brener , D. B. Burckel , R. Narayan , R. Polsky , Adv. Healthcare Mater. 2014, 3, 876. 10.1002/adhm.201300541 24376147

[212] B. Derkus , Biosens. Bioelectron. 2016, 79, 901. 26800206 10.1016/j.bios.2016.01.033

[213] J. Park , Y. Jeong , J. Kim , J. Gu , J. Wang , I. Park , Biosens. Bioelectron. 2020, 148, 111822. 31698304 10.1016/j.bios.2019.111822

[214] S. Yoon , J. K. Sim , Y.‐H. Cho , Sci. Rep. 2016, 6, 23468. 27004608 10.1038/srep23468 PMC4804278

[215] D. J. K. Sim , S. M. Kim , S. S. Kim , I. Doh , Sensors 2019, 19, 3857. 10.3390/s19183857 PMC6767198 31500135

[216] J. K. Sim , B. Ahn , I. Doh , ACS Sens. 2018, 3, 2246. 30354079 10.1021/acssensors.8b00272

[217] X. F. Teng , Y. T. Zhang , Physiol. Meas. 2004, 25, 1323. 15535195 10.1088/0967-3334/25/5/020

[218] J. K. Sim , S. Youn , Y.‐H. Cho , J. Micromech. Microeng. 2012, 22, 125014.

[219] L. K. Randeniya , P. J. Martin , Carbon 2013, 60, 498.

[220] K. Chikkadi , M. Muoth , C. Roman , M. Haluska , C. Hierold , Beilstein J. Nanotechnol. 2014, 5, 2179. 25551046 10.3762/bjnano.5.227 PMC4273237

[221] A. Falco , A. Rivadeneyra , F. C. Loghin , J. F. Salmeron , P. Lugli , A. Abdelhalim , J. Mater. Chem. A 2018, 6, 7107.

[222] K. Chikkadi , M. Muoth , V. Maiwald , C. Roman , C. Hierold , Appl. Phys. Lett. 2013, 103, 223109.

[223] L. Yang , N. Yi , J. Zhu , Z. Cheng , X. Yin , X. Zhang , H. Zhu , H. Cheng , J. Mater. Chem. A 2020, 8, 6487.

[224] J. K. Sim , Y. H. Cho , Sens. Actuators, B 2016, 234, 176.

[225] J. K. Sim , S. Yoon , Y. Cho , Sci. Rep. 2018, 8, 1181. 29352237 10.1038/s41598-018-19239-8 PMC5775419

[226] V. Jain , M. Ochoa , H. Jiang , R. Rahimi , B. Ziaie , Microsyst. Nanoeng. 2019, 5, 29. 31240108 10.1038/s41378-019-0067-0 PMC6572848

[227] A. J. Bandodkar , J. Choi , S. P. Lee , W. J. Jeang , P. Agyare , P. Gutruf , S. Wang , R. A. Sponenburg , J. T. Reeder , S. Schon , T. R. Ray , S. Chen , S. Mehta , S. Ruiz , J. A. Rogers , Adv. Mater. 2019, 31, 1902109. 10.1002/adma.201902109 31206791

[228] D. G. Mackanic , T.‐H. Chang , Z. Huang , Y. Cui , Z. Bao , Chem. Soc. Rev. 2020, 49, 4466. 32483575 10.1039/d0cs00035c

[229] P. Bocchetta , D. Frattini , S. Ghosh , A. M. V. Mohan , Y. Kumar , Y. Kwon , Materials 2020, 13, 2733. 10.3390/ma13122733 PMC7345738 32560176

[230] C. Wu , A. C. Wang , W. Ding , H. Guo , Z. L. Wang , Adv. Energy Mater. 2019, 9, 1802906.

[231] M. Safaei , H. A. Sodano , S. R. Anton , Smart Mater. Struct. 2019, 28, 113001.

[232] S. R. Krishnan , C.‐J. Su , Z. Xie , M. Patel , S. R. Madhvapathy , Y. Xu , J. Freudman , B. Ng , S. Y. Heo , H. Wang , T. R. Ray , J. Leshock , I. Stankiewicz , X. Feng , Y. Huang , P. Gutruf , J. A. Rogers , Small 2018, 14, 1870226. 10.1002/smll.201803192 30369049

[233] A. E. De Paepe , J. Sierpowska , C. Garcia‐Gorro , S. Martinez‐Horta , J. Perez‐Perez , J. Kulisevsky , N. Rodriguez‐Dechicha , I. Vaquer , S. Subira , M. Calopa , E. Muñoz , P. Santacruz , J. Ruiz‐Idiago , C. Mareca , R. de Diego‐Balaguer , E. Camara , Nat. Med. 2020, 26, 418.32161411

[234] S. Park , S. W. Heo , W. Lee , D. Inoue , Z. Jiang , K. Yu , H. Jinno , D. Hashizume , M. Sekino , T. Yokota , K. Fukuda , K. Tajima , T. Someya , Nature 2018, 561, 516. 30258137 10.1038/s41586-018-0536-x

[235] X. Peng , K. Dong , C. Ye , Y. Jiang , S. Zhai , R. Cheng , D. Liu , X. Gao , J. Wang , Z. L. Wang , Sci. Adv. 2020, 6, 9624. 10.1126/sciadv.aba9624 PMC7319766 32637619

[236] Y. Yu , J. Nassar , C. Xu , J. Min , Y. Yang , A. Dai , R. Doshi , A. Huang , Y. Song , R. Gehlhar , A. D. Ames , W. Gao , Sci. Rob. 2020, 5, 7946. 10.1126/scirobotics.aaz7946 PMC7326328 32607455

[237] X. Liu , H. Gao , J. E. Ward , X. Liu , B. Yin , T. Fu , J. Chen , D. R. Lovley , J. Yao , Nature 2020, 578, 550. 32066937 10.1038/s41586-020-2010-9

[238] C. S. Park , Y. C. Shin , S. H. Jo , H. Yoon , W. Choi , B. D. Youn , M. Kim , Nano Energy 2019, 57, 327.

[239] S. Yoon , J. K. Sim , Y. H. Cho , J. Microelectromech. Syst. 2016, 25, 388.

[240] J. L. Blackburn , A. J. Ferguson , C. Cho , J. C. Grunlan , Adv. Mater. 2018, 30, 1870072. 10.1002/adma.201704386 29356158

[241] R. Tian , C. Wan , N. Hayashi , T. Aoai , K. Koumoto , MRS Bull. 2018, 43, 193.

[242] Bin Yang , Xingyu Jiang , Xueen Fang , Jilie Kong , Lab Chip 2021, 21, 4285. 34672310 10.1039/d1lc00438g

[243] S. Imani , A. J. Bandodkar , A. M. V. Mohan , R. Kumar , S. Yu , J. Wang , P. P. Mercier , Nat. Commun. 2016, 7, 11650. 27212140 10.1038/ncomms11650 PMC4879240

[244] S. F. Godek , C. Peduzzi , R. Burkholder , S. Condon , G. Dorshimer , A. R. Bartolozzi , J. Athl. Train. 2010, 45, 364. 20617911 10.4085/1062-6050-45.4.364 PMC2902030

[245] J. Toker Review: The Importance of Potassium Supplementation in Endurance Training and Racing, https://www.saltstick.com.

[246] S. A. N. Gowers , V. F. Curto , C. A. Seneci , C. Wang , S. Anastasova , P. Vadgama , G.‐Z. Yang , M. G. Boutelle , Anal. Chem. 2015, 15, 7763. 10.1021/acs.analchem.5b01353 PMC4526885 26070023

[247] G. Valdés‐Ramírez , A. J. Bandodkar , W. Jia , A. G. Martinez , R. Julian , P. Mercier , J. Wang , Analyst 2014, 139, 1632. 24496180 10.1039/c3an02359a

[248] C. A. Morgan , S. Wang , A. Rasmusson , G. Hazlett , G. Anderson , D. S. Charney , Psychosom. Med. 2001, 63, 412. 11382268 10.1097/00006842-200105000-00010

[249] E. Russell , G. Koren , M. Rieder , S. H. M. Van Uum , Ther. Drug Monit. 2014, 36, 30. 24216536 10.1097/FTD.0b013e31829daa0a

[250] J. C. Pruessner , F. Champagne , M. J. Meaney , A. Dagher , J. Neurosci. 2004, 24, 2825. 15028776 10.1523/JNEUROSCI.3422-03.2004 PMC6729514

[251] Y. Zhao , Y. Gao , D. Zhan , H. Liu , Q. Zhao , Y. Kou , Y. Shao , M. Li , Q. Zhuang , Z. Zhu , Talanta 2005, 66, 51. 18969961 10.1016/j.talanta.2004.09.019

[252] G. Palacios , R. Pedrero‐Chamizo , N. Palacios , B. Maroto‐Sánchez , S. Aznar , M. González‐Gross , Nutr. Hosp. 2015, 31, 237. 25719791 10.3305/nh.2015.31.sup3.8771

[253] R. W. Keller , J. L. Bailey , Y. Wang , J. D. Klein , J. M. Sands , Physiol. Rep. 2016, 4, 12825. 10.14814/phy2.12825 PMC4908500 27273880

[254] M. Huang , X. Pang , R. Letourneau , W. Boucher , T. C. Theoharides , Cardiovasc. Res. 2002, 55, 150. 12062718 10.1016/s0008-6363(02)00336-x

[255] J. A. Jackson , H. D. Riordan , S. Neathery , C. Revard , J. Orthomol. Med. 1998, 13, 236.

[256] J. A. Jackson , H. D. Riordan , S. Neathery , N. H. Riordan , J. Orthomol. Med. 1997, 12, 96.

[257] D. Hirsch , Z. Zukowska , Cell. Mol. Neurobiol. 2012, 32, 645. 22271177 10.1007/s10571-011-9793-z PMC3492947

[258] K. Ito , M. A. Bahry , Y. Hui , M. Furuse , V. S. Chowdhury , Comp. Biochem. Physiol., Part A: Mol. Integr. Physiol. 2015, 187, 13. 10.1016/j.cbpa.2015.04.010 25933935

[259] K. Abid , B. Rochat , P. G. Lassahn , R. Stöcklin , S. Michalet , N. Brakch , J. F. Aubert , B. Vatansever , P. Tella , I. De Meester , E. Grouzmann , J. Biol. Chem. 2009, 284, 24715. 19620246 10.1074/jbc.M109.035253 PMC2757175

[260] M. P. Kalapos , Med. Hypotheses 1999, 53, 236. 10580530 10.1054/mehy.1998.0752

[261] O. Laakso , M. Haapala , T. Kuitunen , J. J. Himberg , J. Anal. Toxicol. 2004, 28, 111. 15068564 10.1093/jat/28.2.111

[262] W. M. Foster , L. Jiang , P. T. Stetkiewicz , T. H. Risby , J. Appl. Physiol. 1996, 80, 706. 8929619 10.1152/jappl.1996.80.2.706

[263] I. Kushch , B. Arendacká , S. Štolc , P. Mochalski , W. Filipiak , K. Schwarz , L. Schwentner , A. Schmid , A. Dzien , M. Lechleitner , V. Witkovský , W. Miekisch , J. Schubert , K. Unterkofler , A. Amann , Clin. Chem. Lab. Med. 2008, 46, 1011. 18605961 10.1515/CCLM.2008.181

[264] C. Turner , P. Španěl , D. Smith , Physiol. Meas. 2006, 27, 13. 16365507 10.1088/0967-3334/27/1/002

[265] C. A. Riely , G. Cohen , M. Lieberman , Science 1974, 183, 208. 4808857 10.1126/science.183.4121.208

[266] B. M. Ross , S. Shah , M. Peet , Open J. Psychiatr. 2011, 1, 4734.

[267] M. L. Bartoli , F. Novelli , F. Costa , L. Malagrin , L. Melosini , E. Bacci , S. Cianchetti , F. L. Dente , A. Di Franco , B. Vagaggini , P. L. Paggiaro , Mediators Inflammation 2011, 2011, 891752. 10.1155/2011/891752 PMC3136125 21772668

[268] B. Antus , O. Drozdovszky , I. Barta , K. Kelemen , Lung 2015, 193, 597. 25951912 10.1007/s00408-015-9739-1

[269] J. D. Fenske , S. E. Paulson , J. Air Waste Manag. Assoc. 1999, 49, 594. 10352577 10.1080/10473289.1999.10463831

[270] M. Torii , M. Yamasaki , T. Sasaki , H. Nakayama , Br. J. Sports Med. 1992, 26, 29. 1600450 10.1136/bjsm.26.1.29 PMC1478977

[271] S. Yoon , J. K. Sim , Y. H. Cho , Sci. Rep. 2016, 6, 23468. 27004608 10.1038/srep23468 PMC4804278

[272] H. G. Kim , E. J. Cheon , D. S. Bai , Y. H. Lee , B. H. Koo , Psychiatry Investig. 2018, 15, 235. 10.30773/pi.2017.08.17 PMC5900369 29486547

[273] K. Błazejczyk , G. Jendritzky , P. Bröde , D. Fiala , G. Havenith , Y. Epstein , A. Psikuta , B. Kampmann , Geogr. Pol. 2013, 86, 5.

[274] J. Mathew , Y. Semenova , G. Farrell , Biomed. Opt. Express 2012, 3, 3325. 23243581 10.1364/BOE.3.003325 PMC3521315

[275] P. H. Charlton , D. A. Birrenkott , T. Bonnici , M. A. F. Pimentel , A. E. W. Johnson , J. Alastruey , L. Tarassenko , P. J. Watkinson , R. Beale , D. A. Clifton , IEEE Rev. Biomed. Eng. 2018, 11, 2. 29990026 10.1109/RBME.2017.2763681 PMC7612521

[276] M. Trapp , E.‐M. Trapp , J. W. Egger , W. Domej , G. Schillaci , A. Avian , P. M. Rohrer , N. Hörlesberger , D. Magometschnigg , M. Cervar‐Zivkovic , P. Komericki , R. Velik , J. Baulmann , PLoS One 2014, 9, 89005. 10.1371/journal.pone.0089005 PMC4015896 24817135

[277] J. K. Sim , B. Ahn , I. Doh , AIP Adv. 2018, 8, 045210.

